# Zuogui Jiangtang Shuxin formula Ameliorates diabetic cardiomyopathy mice *via* modulating gut-heart axis

**DOI:** 10.3389/fendo.2023.1106812

**Published:** 2023-02-09

**Authors:** Ya-lan Huang, Qin Xiang, Jun-ju Zou, Yongjun Wu, Rong Yu

**Affiliations:** ^1^ The First Hospital of Hunan University of Chinese Medicine, Hunan University of Chinese Medicine, Changsha, China; ^2^ Graduate School, Hunan University of Chinese Medicine, Changsha, China; ^3^ School of Traditional Chinese Medicine, Hunan University of Chinese Medicine, Changsha, China; ^4^ School of Pharmacy, Hunan University of Chinese Medicine, Changsha, China

**Keywords:** Zuogui Jiangtang Shuxin formula, diabetic cardiomyopathy, gut microbiota, gut - heart axis, TMAO/PERK/FOXO1

## Abstract

**Background:**

There is growing evidence demonstrating that the gut microbiota plays a crucial role in multiple endocrine disorders, including diabetic cardiomyopathy (DCM). Research shows that the Chinese herb reduces disease occurrence by regulating gut microbiota. Zuogui Jiangtang Shuxin formula (ZGJTSXF), a Chinese medicinal formula, has been clinically used for treatment of DCM for many years. However, there is still no clear understanding of how ZGJTSXF treatment contributes to the prevention and treatment of DCM through its interaction with gut microbiota and metabolism.

**Methods:**

In this study, mice models of DCM were established, and ZGJTSXF’s therapeutic effects were assessed. Specifically, serum glycolipid, echocardiography, histological staining, myocardial apoptosis rate were assessed. Using 16s rRNA sequencing and high-performance liquid chromatography-tandem mass spectrometry (HPLC-MS/MS), we determined the impact of ZGJTSXF on the structure of gut microbiota and content of its metabolite TMAO. The mechanism of ZGJTSXF action on DCM was analyzed using quantitative real-time PCR and western blots.

**Results:**

We found that ZGJTSXF significantly ameliorated DCM mice by modulating gut-heart axis: ZGJTSXF administration improved glycolipid levels, heart function, cardiac morphological changes, inhibited cardiomyocytes apoptosis, and regulate the gut microbiota in DCM mice. Specifically, ZGJTSXF treatment reverse the significant changes in the abundance of certain genera closely related to DCM phenotype, including Lactobacillus, Alloprevotella and Alistipes. Furthermore, ZGJTSXF alleviated DCM in mice by blunting TMAO/PERK/FoxO1 signaling pathway genes and proteins.

**Conclusion:**

ZGJTSXF administration could ameliorate DCM mice by remodeling gut microbiota structure, reducing serum TMAO generation and suppressing TMAO/PERK/FoxO1 signaling pathway.

## Introduction

Over the past few decades, type 2 diabetes mellitus (T2DM) and its complications have increased annually (1). As a common cardiovascular complication of diabetes, diabetic cardiomyopathy (DCM) primarily manifests as an abnormal myocardium, independent of coronary artery disease, valvular disease, and cardiovascular risk factors such as hypertension (2, 3). A persistent condition in which glucose and lipid metabolism causes cardiomyocyte death, myocardial fibrosis, ventricular remodeling, and diastolic and systolic dysfunction (4). It is one of the primary causes of death among diabetics (5). In spite of intensive efforts, no definitive therapeutic methods have been developed for treating DCM. Furthermore, there are a limited number of treatment options and no understanding of the underlying pathogenesis of essential DCM. As a result, identifying potential therapeutic targets and discovering novel mechanisms of DCM are essential to preventing and treating the disease.

Maintaining a healthy microbiota in the gut is crucial for maintaining homeostasis. However, when gut microbial homeostasis is disrupted, it can induce the development of different diseases. It has recently been found that dysbiosis of the gut microbiota plays a role in multiple conditions, including obesity, metabolic syndrome, diabetes, and cardiovascular disease, which are closely linked to DCM (6). There are many ways in which gut microbiota communicate with heart, including the production of trimethylamine-N-oxide (TMAO), short-chain fatty acids (SCFAs), bile acids, lipopolysaccharide (LPS), and phenylacetylglutamine (PAGln) (7, 8). This intimate connection defines the term gut-heart axis. Numerous cardiovascular disorders have been linked to the gut-heart axis, and addressing the gut-heart axis may ameliorate DCM (9). TMAO is one of the more extensively studied metabolites formed by the gut microbiota and comes with a potential role in cardiovascular diseases (10–12). Plasma TMAO levels are also associated with a significantly higher risk of type 2 diabetes and metabolic syndrome (13). In the context of pathogenesis, further research is needed to better understand how microbiota gut-derived metabolites TMAO communicate with the heart. Research conducted by Chen et al. indicated that TMAO bound and activated PERK (an endoplasmic reticulum stress sensor), which caused FoxO1 to induce insulin resistance and metabolic dysfunction (14). Furthermore, inhibition of TMAO production can reduce the activation of PERK and inhibit FoxO1, which may prevent hyperglycemia (14, 15). Converging evidences above, we postulated that TMAO/PERK/FoxO1 signaling pathway is possibly a potent target for the treatment of DCM. The above-mentioned ideas are only our reasonable speculation, and the specific mechanism needs to be more thoroughly investigated.

Over 2,000 years of history have been devoted to traditional Chinese medicine (TCM), which is widely used to treat metabolic and cardiovascular disorders. Recently, gut microbiota has emerged as an invaluable field for understanding TCM (16). There is compelling evidence that TCM may influence gut microbiota and metabolic components through interactions with gut microbiota (17). The Zuogui Jiangtang Shuxin formula (ZGJTSXF), a herbal compound based on meridian theory, is a herb that addresses a wide range of health conditions. The ZGJTSXF has been widely used in clinical practice for the treatment of diabetic cardiovascular complications. This is due to its effectiveness in nourishing Yin and benefiting Qi, invigorating blood, and removing toxins. Although studies have shown the efficacy of ZGJTSXF in DCM, the mechanism of action of ZGJTSXF in the treatment of DCM are not fully understood, which limits the further development and clinical application of ZGJTSXF. In light of this, further investigation of ZGJTSXF in terms of the prevention and treatment of DCM upon gut microbiota-mediated insight may contribute to the understanding of its mechanism of action and the potential clinical applications of the compound.

Here, the effects of ZGJTSXF treatment on mice models of DCM were evaluated first. We investigated the shifts in gut microbiota and content of its metabolite TMAO using 16S rRNA gene sequencing and HPLC-MS/MS. To determine the potential therapeutic effects of ZGJTSXF on the gut-heart axis, a holistic correlation analysis uniting gut microbiome and metabolomics was conducted. Throughout the whole process, our results presented substantial evidence about how ZGJTSXF ameliorated DCM by reshaping gut microbiota and modifying metabolites. This provided more theoretical and experimental proof of gut microbiota as an essential factor in diseases.

## Materials and methods

### Preparation and component analysis of ZGJTSXF

Herbs in ZGJTSXF [composed of Panax ginseng C. A. Meyer, Astragalus membranaceus (Fisch.), Bunge, Rehmannia glutinosa (Gaetn.) Libosch. ex Fisch. et Mey., Pueraria lobata (Willd.), Ohwi, Cornus officinalis Sieb. et Zucc., Salvia miltiorrhiza Bunge, Coptis chinensis Franch., Ophiopogon japonicus (Linn. f.) Ker-Gawl. and Crataegus pinnatifida Bge] were provided by herbal pharmacy of First Hospital of Hunan University of Chinese Medicine (Hunan, China) and authenticated by Professor Professor Yong-jun Wu from School of Pharmacy of Hunan University of Chinese Medicine. We weighed each medicinal material accurately and soaked it in distilled water for one hour. The drugs were boiled twice in water, each time for one hour. A water decoction containing the 2 g·mL−1 original medicinal material was prepared from the double-extracted solutions using filtration, concentration, and packaging. The resulting decoction was stored in a refrigerator (4°C) until it was used.

Take 15 ml of herbal decoction, centrifuge at a high speed (12,000 rpm, 15 min), and analyze the supernatants using ultra high performance liquid chromatography - high resolution mass spectrometry (UPLC-Q-Exactive-Orbitrap-MS). UPLC-Q-TOF/MS grade acetonitrile and HPLC grade acetonitrile, methanol, formic acid, were provided by Merck KGaA (Darmstadt, Germany). UPLC-Q-Exactive-Orbitrap-MS analysis was analyzed on a Waters Corporation Xbridge BEH C18 (2.1 mm×100 mm, 2.6 m) system, which was maintained at 40°C. The flow rate was set at 0.3mL/min, and the injection volume was 10 µL. The mobile phase was consisted of 0.1% formic acid water (A) - 0.1% formic acid acetonitrile (B) (0~1.5 min, 2%~2% B; 1.5~20 min, 2%~45% B; 20~27 min, 45%~95% B; 27~32 min, 95%~95% B; 32~32.1 min, 95%~2% B; 32.1~35 min, 2%~2% B). The eluent was detected by a quadrupole orbitrap high resolution mass spectrometer in the ESI positive and negative ion mode. The raw data were processed using Xcalibur 4.3 and Compound Discoverer 3.2 software (Thermo Fisher Scientific, USA).

### Experimental animals and study design

The MKR mice, which were first established by Fernandez and colleagues and bear a dominant-negative IGF-1R in skeletal muscle (18), were obtained from Dr. Derek LeRoith in National Institutes of Health Diabetes Research Center (Bethesda, MD, USA). MKR mice were housed in a temperature (22 ± 2 °C) controlled room with a 12 h light/dark cycle. MKR mice were used for breeding and their offspring were used for experiments.

Twelve male MKR mice (8 weeks old) were randomly divided into two groups: 1) diabetic cardiomyopathy model group (DCM, n = 6); 2) ZGJTSXF treatment group (ZGJTSXF, n = 6). Both groups were kept on a high-fat diet supplemented with 1% choline for four weeks. After four weeks, MKR mice were injected with 1% streptozotocin (STZ; Sigma Aldrich Co., USA) dissolved in citrate buffer (pH=4.5) at a dose of 40 mg/kg/day for 5 days. Fasting blood glucose (FBG) values and echocardiography were tested to determine the development of diabetes in desired groups. Age-matched and sex-matched nondiabetic normal C57BL/6 mice were used as control group (CON, n = 6). Our previous study explored the efficacy of three doses (16.84 g/kg/d, 33.67 g/kg/d, and 67.34 g/kg/d) of this prescription, and the result demonstrated that ZGJTSXF owns optimal efficacy when it is administered at 33.67 g/kg/d; therefore, a dose of 33.67 g/kg/d was chosen for the experiments in the current study (unpublished observations). The mice in the CON and DCM groups were given equal volumes of distilled water. All groups were gavaged once a day for 4 weeks. The protocols for animal care and handling were approved by the Animal Ethical Committee of the Hunan University of Chinese Medicine.

### Serum glycolipid profile

The mice were fasted for eight hours after the last administration. The tail vein was then accessed for blood collection. Blood glucose levels were measured with a blood glucose monitor (GT-1980. Aikelai Medical Electronics (Pinghu) Co., Ltd., China). An oral glucose tolerance test (OGTT) was conducted on mice after fasting for 8 hours (free access to water). A blood glucometer measured blood glucose levels in tail veins after glucose loading at 0, 30, 60, 90, and 120 minutes. An area under the curve (AUC) for glucose was calculated using the trapezoidal method based on five glucose measurements. Following an eight-hour fast, mice were anesthetized with light isoflurane anesthesia and blood was drawn from the retroorbital plexus. Blood samples were processed immediately, centrifuged and frozen at −80 °C until assayed. Fasting serum insulin (FINS) was determined according to the manufacturer’s instructions using an enzyme linked immunosorbent assay (ELISA) kit (Wuhan, China). Insulin resistance index (HOMA-IR) was estimated according to the formula: HOMA-IR=FBG (mmol/L)×FBI (mIU/mL)/22.5. The serum total cholesterol (TC) and serum triglycerides (TG) levels as well as the high density lipoprotein cholesterol (HDL-C) and serum low density lipoprotein cholesterol (LDL-C) levels were determined using commercially available kits (Nanjing Jiancheng Bioengineering Institute, Nanjing, China).

### Echocardiography analysis

We anesthetized the animals with 1.5% isoflurane in 95% oxygen and 5% carbon dioxide, and removed their chest hair with a depilatory cream before examination. We assessed *in vivo* heart function using a high-resolution ultrasound imaging system (VINNO 6, Vinno Corporation, Suzhou, China) and measured chamber dimensions with a 23 MHz frequency transducer. An M-mode recording was obtained from short-axis parasternal views. We measured and recorded the internal dimensions of ejection fraction (EF), fractional shortening (FS), left ventricular end-systolic diameter (LVIDs), left ventricular end-diastolic diameter (LVIDd), left ventricular end-systolic volume (LVEDV), and left ventricular end-diastolic volume (LVEDV). A reading average is calculated based on at least three measurements in echocardiography.

### Histological analysis

Heart tissues were fixed for 24 hours in 0.01 M phosphate-buffered saline and 10% formalin. A slide was prepared by embedding fixed tissues in paraffin and separating them into thin sections of 5 um thickness. The slides were stained with hematoxylin and eosin (H&E), Picrosirius Red Stain Kit (Wellbio, Changsha, China), and Masson’s trichrome stain Kit (Wellbio, Changsha, China) for histopathological comparisons and determined by the light microscopy (Motic China Group Co., Ltd.).

### Terminal deoxynucleotidyl transferase dUTP nick end labeling staining

As needed, TdT and dUTP reagents from the TUNEL kit were mixed at a ratio of 1:5 according to the sample size. Sections were incubated in a water bath at 37°C for 60 minutes after being covered and placed in a wet box. A dropwise application of 4’, 6-diamidino-2-phenylindole stain was applied to the slides after they had been rinsed three times in PBS for 5 minutes each. New glass slides were used for sealing the cells, as well as anti-fluorescence quenching tablets. Photographs were taken of the slides under a fluorescence microscope (BX53, OLYMPUS, Japan). Apoptotic cells fluoresce red. MicroPublisher imaging system (Q-imaging) was used to calculate AI using five visual fields of each tissue. The AI calculation was based on the following equation: AI = apoptotic nuclei/total cardiac nuclei.

### Gut microbial analysis of cecal contents

Each cecal content sample was extracted using HiPure Stool DNA Kit B (Magen, Shanghai, China) following the manufacturer’s instructions and quantified by ultraviolet spectroscopy. The 16S rDNA V3-V4 region was amplified by PCR (94°C for 2 min, followed by 30 cycles at 98°C for 10 s, 62°C for 30 s and 68°C for 30 s and a final extension at 68°C for 5 min) using universal forward and reverse primers 341F (CCTACGGGNGGCWGCAG) and 806R (GGACTACHVGGGTATCTAAT). AxyPrep DNA Gel Extraction Kit (Axygen Biosciences, Union City, CA, US) was used to purify amplicons from 2% agarose gels. Life Technologies, Foster City, USA) provided the ABI StepOnePlus Real-Time PCR System for quantification. An illumina sequencing platform was used to sequence paired-end amplicons pooled in equimolar concentrations according to Gene Denovo Biotechnology Co. Ltd (Guangzhou, China). Raw reads were further filtered using FASTP (version 0.18.0). Raw tags were merged using FLASH (version 1.2.11) with a minimum overlap of 10 bp and a 2% mismatch error rate. Quality filtering and removal of chimeric sequences were then performed before determining which tags are effective, and then clustering them according to the ≥97% similarity cutoff using UPARSE software (version 9.2.64). An analysis of principal components (PCA) was conducted using Vegan R (version 2.5.3). In order to analyze the effect of the DCM and ZGJTSXF on the overall microbiota structure, we used QIIME software (version 1.9.1, University of Colorado, Denver, CO, USA) to obtain observed species, abundance-based coverage estimator (ACE), Shannon diversity index, and Simpson diversity index. Utilizing the R project, we analyzed bacteria mainly at the phylum and genus level. In the Vegan package (version 2.5.3) of the R project, Welch’s t-test was applied to compare species between groups. In addition, the linear discriminant analysis (LDA) effect size measurement (LefSe) analysis based on Kruskal-Wallis rank-sum test and Tukey HSD test was conducted to identify the abundant taxonomy with significant differences among the three groups. In order to calculate the heat map of cluster stacking, we used R and Omicsmart (Gene Denovo Biotechnology Co. Ltd, Guangzhou, China), a dynamic real-time interactive platform for data analysis.

### Serum TMAO detected by HPLC-MS/MS

In this study, serum TMAO was measured by high-performance liquid chromatography-tandem mass spectrometry (HPLC-MS/MS). The sample was prepared by adding 10 uL of TMAO d9 (Toronto Research Chemicals Inc., Toronto, Canada) to 100 uL serum; 300 uL of acetonitrile precipitated the protein, vortexed for 1 min, centrifuged at 1,000 rpm, 4°C for 5 min; and 200 uL of the remaining supernatant was injected into a Waters Atlantis HILIC Silica column for analysis.

### Real-time quantitative PCR analysis

The total RNA of cells and tissues was extracted using the Total RNA Extracting Kit (Foregene Co. Ltd., China). Total RNA was reverse-transcribed to synthesize single strand complementary DNA (cDNA) using RT EasyTM II (with gDNase) (RT-01032) kit (Foregene Co. Ltd., China). Real Time PCR EasyTM-SYBR Green I (QP-01014; Foregene Co. Ltd., China) and LightCycler 96 Instrument (Roche, Mannheim, Germany) were used for real-time quantitative PCR (qPCR). GAPDH was used as the internal reference gene for qPCR, and gene expression levels were calculated with the 2^-ΔΔ^CT method. The primers of each gene are as follows: Forward-PERK, 5’-CAGTGTTTGGCTTAGGGGCA-3; Reverse-PERK, 5’-TCATTCTCGGCATCCAGTGC-3; Forward-FoxO1, 5’-TTTCGTCCTCGAACCAGCTC-3; Reverse-FoxO1, 5’- TACACCAGGGAATGCACGTC-3; Forward-Bim, 5’-AAATGGCCAAGCAACCTTCTG-3’; Reverse-Bim, 5’- CTTGCGGTTCTGTCTGTAGGG-3’; Forward-Puma, 5’-TGGGAGATATTGGCGGAAGC-3’; Reverse-Puma,5’- GTATCTTACAGGCTGGGCCG-3’; Forward-TNFSF10, 5’-GGAAGACCTCAGAAAGTGGCA-3’; Reverse-TNFSF10, 5’- CTCGATGACCAGCTCTCCATT-3’;

Forward-GAPDH, 5’-ACTCTTCCACCTTCGATGCC-3; Reverse-GAPDH, 5’-TGGGATAGGGCCTCTCTTGC-3’.

### Western blot analysis

A homogenized heart tissue sample was centrifuged at 12,000 rpm at 4°C for 10 minutes in lysis buffer (Biyuntian Biotech Co. Ltd., China). The protein-containing supernatant was collected. The protein concentrations were determined using the BCA (Bicinchoninic Acid) protein assay kit from Biyuntian Company. Proteins were separated by 10% sodium dodecyl-sulfate polyacrylamide gel electrophoresis (SDS-PAGE) and transferred to polyvinylidene difluoride membranes (PVDF). After soaking in TBST buffer supplemented with 5% bovine serum albumin (BSA) for 1 h, these PVDF membranes were incubated with primary antibodies overnight at 4°C. It was then washed three times with TBST and incubated at room temperature for 1 hour with secondary antibodies conjugated to horseradish peroxidase (HRP). Rewashed with TBST, the membranes were imaged on X-ray film by chemiluminescence. The super-sensitive ECL chemiluminescent substrate kit was purchased from Biosharp Life Science Co., Ltd. (China). The band intensity was analyzed using ImageJ software (National Institutes of Health, Maryland, USA). The primary antibodies used in this study were as follows: FOXO1 Antibody (AF301660), FoxO1 (phospho Ser256) Polyclonal Antibody (AF00557), Bim Antibody (AF300342), Bim (phospho Ser59) Polyclonal Antibody (AF00289), PUMA Antibody (AF300458) were provided by AiFang biological (Hunan, China), Phospho-PERK (Thr982) Antibody (DF7576) was purchased from Affinity Biosciences (Jiangsu, China), Anti -TRAIL Rabbit pAb (GB11413) were provided by Sevier Biotechnology Co., Ltd. (Wuhan, China)., Anti-PERK antibody [EPR19876-294] (ab229912) and anti-GAPDH antibody [EPR16891] (ab181602) were supplied by Abcam Biotech (Shanghai, China).).

### Statistical analysis

We express all data as mean ± standard deviation. When the measurement data conformed to normal distribution and the homogeneity of variance test was homogeneous, one-way ANOVA was used to compare groups, whereas paired samples t-tests were used to compare before-and-after data. Otherwise, the Kruskal-Wallis test and the Wilcoxon rank test are used. *P*<0.05 was considered statistically significant. SPSS 22.0 (IBM, USA) was used for all analyses.

## Results

### Major components in ZGJTSXF according to UPLC-Q-exactive-orbitrap-MS analysis

Based on the established UPLC-Q-Exactive-Orbitrap-MS method, 290 Chemical compounds were identified. These components can be divided into ten categories: flavonoids, acids/small peptides, phenylpropanoids, phenols, saponins, organic acids, alkaloids, nucleosides, iridoids and oligoses. In this study, the chromatogram and detailed composition of ZGJTSXF are shown in **Supplementary Figure S1** and **Table S1**.

### ZGJTSXF administration improves glucose and lipid metabolism in DCM mice

**Figure 1:**
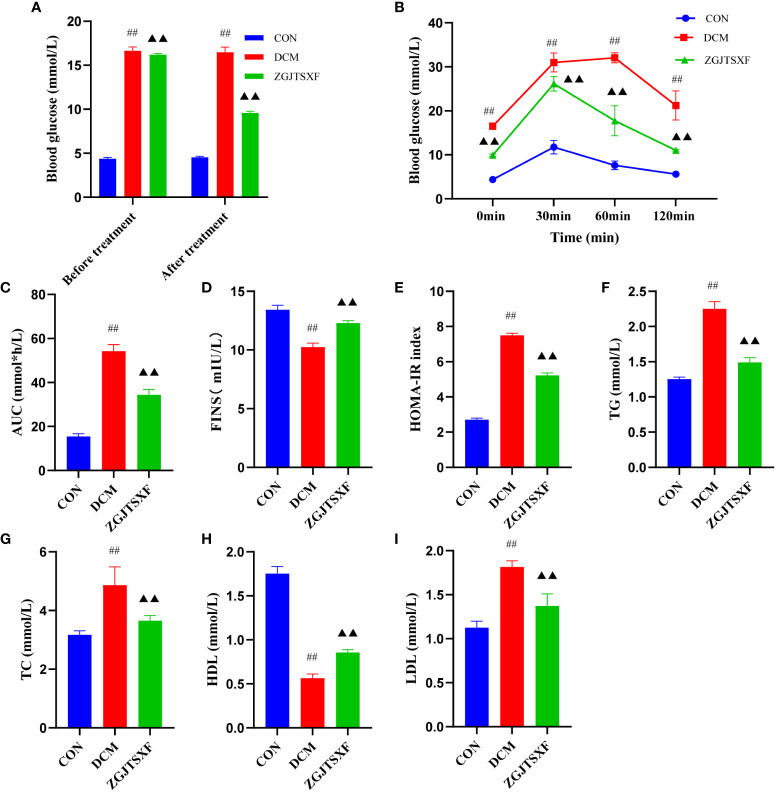
ZGJTSXF administration can improve glucose and lipid metabolism. **(A)** Fasting blood glucose levels before and after treatment in all groups of mice.**(B)** Oral glucose tolerance test (OGTT) levels of each group at different time periods. **(C)** The area under the curve (AUC) of plasma glucose during OGTT. **(D)** Fasting serum insulin (FINS). **(E)** Insulin resistance index (homeostasis model assessment of insulin resistance, HOMA-IR). **(F)** Triglycerides(TG). **(G)** Total Cholesterol (TC). **(H)** High-Density lipoprotein Cholesterol (HDL). **(I)** low-Density lipoprotein Cholesterol. ^##^ *P*<0.01, compared with the CON group; ^▲▲^ *P*<0.01, compared with the DCM group.

First, we evaluated the impacts of ZGJTSXF administration on FBG in mice with DCM. The results showed that the FBG levels of the DCM group were significantly higher than those of the CON group (*P*<0.01). After 4 weeks of intervention, compared with the DCM group, the ZGJTSXF group showed significantly reduced serum FBG levels in DCM mice (*P*<0.01) (**Figure 1A**). Therefore, we confirmed that ZGJTSXF administration did exhibit significant benefits in terms of lowering FBG.

Next, we evaluated the impacts of ZGJTSXF administration on oral glucose tolerance in mice with DCM. As shown in **Figure 1B** and **Figure 1C**, after the intake of glucose, compared with the CON group, the DCM group had significantly increased blood glucose level at each time point (*P*<0.01), while the ZGJTSXF group after treatment significantly alleviated the blood glucose increase in DCM mice (*P*<0.01).

we also evaluated the impacts of ZGJTSXF administration on fasting serum insulin (FINS) in mice with DCM. As shown in **Figure 1**, The results showed that the FINS levels of the DCM group were significantly higher than those of the CON group (*P*<0.01)(**Figure 1D**). After 4 weeks of intervention, compared with the DCM group, the ZGJTSXF group showed significantly reduced FINS levels in DCM mice (*P*<0.01). Due to its good correlation to glycemic clamp, HOMA-IR has been widely utilized as insulin resistance index in clinical and epidemiological studies (19, 20). The results showed that the insulin resistance index were increased in the DCM group compared with the CON group (*P*<0.01), whereas lower insulin resistance index were found in the ZGJTSXF group compared with that in the DCM group (*P*<0.01) (**Figure 1E**).

In addition, the antihyperlipidemic effect of ZGJTSXF in DCM mice has also been emphasized in this experiment. As shown in **Figure 1**, DCM group showed significant increased serum TG, TC, and LDL-C and decreased serum HDL-C compared with the CON group (*P*<0.01). Administration of ZGJTSXF reduced serum TG (**Figure 1F**), TC (**Figure 1G**), LDL-C (**Figure 1H**) and increased HDL-C in diabetic mice (**Figure 1I**) (*P*<0.01).

### ZGJTSXF administration promoted myocardial function and myocardial histology in DCM mice

**Figure 2:**
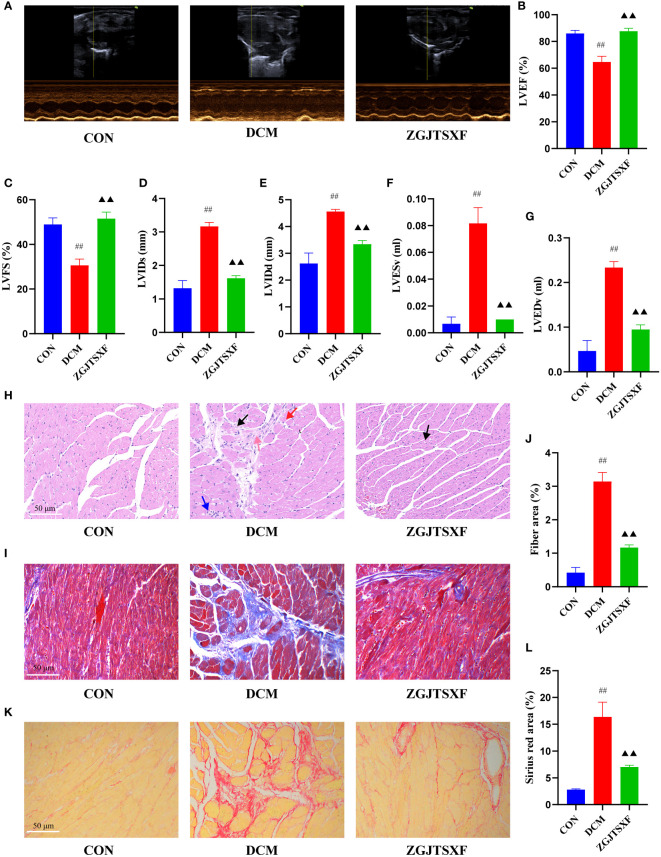
ZGJTSXF administration changed myocardial function and myocardial histology in DCM mice. **(A–G)** Echocardiographic observation of changes in cardiac function in various groups of mice. **(A)** Representative M-mode echocardiograms in mice of the indicated groups. **(B)** Summary on the left ventricle fractional shortening (LVFS). **(C)** Summary on the left ventricle ejection fraction (LVEF). **(D)** Summary on left ventricular internal end-systolic diameter (LVIDs). **(E)** Summary on left ventricular internal end-diastolic diameter (LVIDd). **(F)** Summary on left ventricular end-systolic volume (LVESV). **(G)**Summary on left ventricular end-diastolic volume (LVEDV). **(H)** Representative images of a mice myocardial tissues after HE staining (Scale bar, 50 μm). **(I)** Representative images of mice myocardial tissues after masson staining (Scale bar, 50 μm). **(J)** Fiber rate of cardiomyocytes in mice of the indicated group was summarized. **(K)** Representative images of a mice myocardial tissues after sirius red staining (Scale bar, 50 μm). **(L)** Sirius red area of cardiomyocytes in mice of the indicated group was summarized. Black arrow, vacuolar degeneration of cardiomyocytes; red arrow, coagulative necrosis of the cardiomyocytes; blue arrow, small focal inflammatory cell infiltrates in the interstitial myocardium; pink arrow, myocardial fibrosis. ^##^ *P*<0.01, compared with the CON group; ^▲▲^ *P*<0.01, compared with the DCM group.

We evaluated the effects of ZGJTSXF on echocardiography in DCM mice. As shown in **Figure 2A**, echocardiography analysis found that mice in DCM group had significantly reduced left ventricle ejection fraction (LVEF) and left ventricle fractional shortening (LVFS), compared with mice in CON group. Notably, mice ZGJTSXF groups showed significantly increased LVEF and LVFS (**Figures 2B–C**) (*P*<0.01). The left ventricular internal end-systolic diameter (LVIDs), left ventricular internal end-diastolic diameter (LVIDd), left ventricular end-systolic volume (LVESV) and left ventricular end-diastolic volume (LVEDV) of mice in the DCM group significantly increased compared to those of mice in the CON groups (*P*<0.01). On the contrary, the ZGJTSXF group exhibited significantly reduced left ventricular internal end-systolic diameter (LVIDs), left ventricular internal end-diastolic diameter (LVIDd), left ventricular end-systolic volume (LVESV) and left ventricular end-diastolic volume (LVEDV) compared with the DCM group (*P*<0.01) (**Figures 2D–G**).

We further checked how ZGJTSXF administration could influence the histological changes in myocardial tissues. As shown in **Figure 2H**, in the CON group of mice, the ventricular wall and papillary muscle cells were arranged neatly and regularly; the nuclei were of uniform size; the cell gaps were not widened or narrowed; the morphology and structure were good. No pathological changes were detected in the CON group. In the DCM group, there was vacuolar degeneration of cardiomyocytes, coagulative necrosis of the cardiomyocytes, small focal inflammatory cell infiltrates in the interstitial myocardium and myocardial fibrosis. However, coagulative necrosis of the cardiomyocytes, small focal inflammatory cell infiltrates in the interstitial myocardium and myocardial fibrosis were significantly improved in the ZGJTSXF groups, compared to the DCM group.

Cardiovascular fibrosis contributes to the pathogenic remodeling and structural changes of diabetic hearts (21), which further contributes to DCM myocardial dysfunction. We investigated whether ZGJTSXF could inhibit cardiac fibrosis synthesis. As a major method for assessing cardiac fibrosis, Masson staining uses collagen deposition to determine collagen deposition. **Figures 2I, J** show that collagen was practically absent in the CON group, whereas collagen clumps accumulated in the DCM group (*P* < 0.01). There was an improvement in cardiac fibrosis in the ZGJTSXF treatment group (*P* < 0.05). Sirius red staining reacts with collagen fibers to test collagen deposition. As shown in **Figure 2K** and **Figure 2L**, no significant collagen was observed in the CON group, but significant increase collagen appeared in DCM group (*P <*0.01). However, ZGJTSXF administration remarkably decreased collagen content in DCM mice (*P <*0.01).

### ZGJTSXF administration inhibited apoptosis in DCM mice

**Figure 3:**
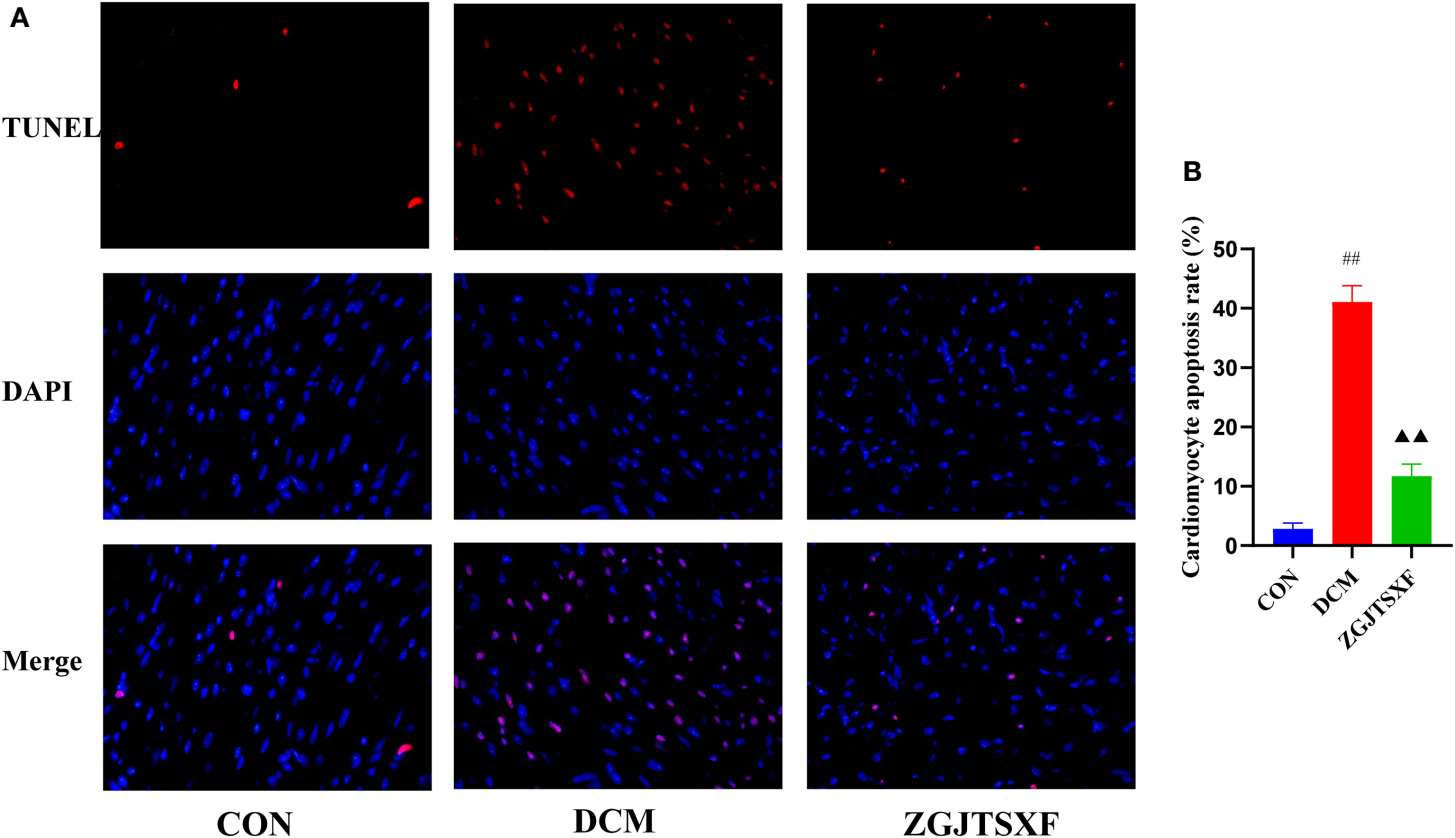
Apoptosis of mouse myocardial tissues was evaluated by TUNEL staining. **(A)** Representative images of apoptosis of mice myocardial tissues after TUNEL staining. **(B)** Apoptosis rate of cardiomyocytes in mice of the indicated group was summarized. ^##^ *P*<0.01, compared with the CON group; ^▲▲^ *P*<0.01, compared with the DCM group.

We quantitated the apoptosis of mouse cardiomyocytes through TUNEL assay. As shown in **Figure 3A** and **Figure 3B**, the apoptosis rate of cardiomyocytes in the DCM group was significantly higher than that in the CON group (*P <*0.01). Compared with the DCM group, the ZGJTSXF groups demonstrated significantly reduced cardiomyocyte apoptosis (*P <*0.01).

### ZGJTSXF administration affected the structure of the gut microbiota in DCM mice

Intestinal microbes have been recognized to play an important role in ameliorating DCM. Studies indicated that oral drug administration may influence disease development *via* modulating composition and metabolites of gut microbiota (22). To this end, gut microbiota were investigated in animals after oral administration with ZGJTSXF.

**Figure 4:**
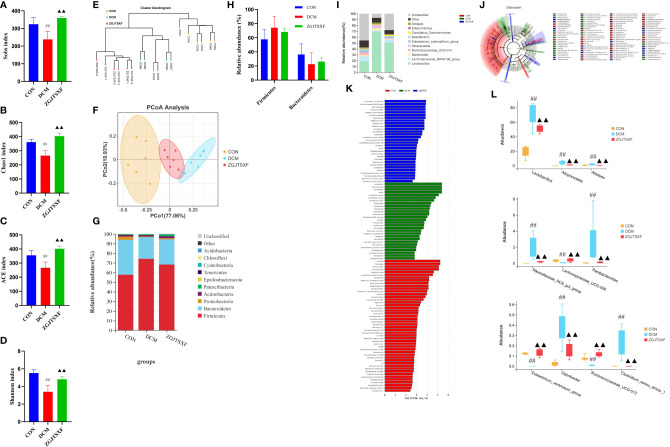
ZGJTSXF administration changed the structure of the gut microbiota in DCM mice. **(A–D)** Analysis of α diversity of gut microbiota in mice. **(A)** Sobs 1 index. **(B)** Chao 1 index. **(C)** ACE index. **(D)** Shannon index. **(E, F)** Analysis of β diversity of gut microbiota in mice. **(E)** UPGMA clustering tree and **(F)** principal coordinates analysis (PCoA) based on Jaccard distance. **(G)** The overall composition of the gut microbiota at the phylum level. **(H)** The relative abundance of Firmicutes and Bacteroidetes. **(I)** The overall composition of the gut microbiota at the Genus level. **(J)** Cladogram generated by the LEfSe analysis **(K)** Linear discriminant analysis (LDA) score for taxa differing between three groups. An LDA score greater than 2 indicated a higher relative abundance in the corresponding group than in the three groups. Blue bars represent taxa that are significantly increased in the DCM group. Green bars represent taxa that are significantly increased in the ZGJTSXF group. Red bars represent taxa that are significantly increased in the CON group. **(L)** The relative abundance of Lactobacillus, Alloprevotella, Alistipes, Rikenellaceae_RC9_gut_group, Lachnospiraceae_UCG-006, Parabacteroides, Eubacterium_ventriosum_group, Odoribacter, Ruminococcaceae_UCG-013, Clostridium_sensu_stricto_1. ^##^ *P*<0.01, compared with the CON group; ^▲▲^ *P*<0.01, compared with the DCM group.

The gut microbiota diversity and richness were evaluated by Sobs, Chao1, ACE and Shannon indexes. Compared with the control and ZGJTSXF treatment groups (**Figures 4A–D**), relatively few bacterial species were seen in the DCM groups (*P* < 0.05, *P* < 0.001), indicating that ZGJTSXF is altering bacterial community abundance. β-diversity analysis was used to assess the discrepancies between microbial communities. UPGMA clustering tree and principal coordinates analysis (PCoA) based on Jaccard distance were used to analyze changes in the overall structure of gut microbiota (**Figures 4E, F**). Using UPGMA and PCoA, it was evident that DCM and CON were aggregated separately. This suggested that the CON group samples and the DCM model group samples had different compositions and structures. In contrast, the distance between the ZGJTSXF treatment group and the CON group was closer than that between the DCM model group and the CON treatment group. These results suggested that ZGJTSXF altered the diversity reduction of intestinal microorganisms and the structure of microbial communities challenged by DCM.

We determined the relative abundance of the phylum (**Figure 4G**) and found that the DCM mice displayed an increased the relative abundance of Firmicutes (74.21% vs. 57.70%) and decreased abundance of Bacteroidetes (22.50% vs. 36.33%) compared with those in the CON group. In contrast, the microbiota imbalance was ameliorated by ZGJTSXF administration as it decreased the abundance of Firmicutes (68.29% vs. 74.21%) and increased Bacteroidetes (26.09% vs. 22.51%) (**Figure 4H**). Furthermore, treatment with ZGJTSXF reduced the ratio of Firmicutes to Bacteroidetes in DCM mice, although the difference was not statistically significant (**Supplementary Figure S2**). A decrease in Bacteroidetes or an increase in Firmicutes or an increase in the ratio of Firmicutes to Bacteroidetes contributes to the risk of diabetes (23). Therefore, this result showed that the ZGJTSXF could reduce the risk of diabetes.

At the genus level (**Figure 4I**), DCM mice had a higher relative abundance of Lactobacillus, Bacteroides, Alloprevotella and Alistipes and had a lower relative abundance Lachnospiraceae_NK4A136_group, Ruminococcaceae_UCG-014, Eubacterium_xylanophilum_group and Desulfovibrio compared with those of CON mice. However, the abundance of these bacteria in the ZGJTSXF group were reversed and returned to CON group compared with the DCM group.

The LEfSe analysis, which emphasizes the statistical significance and biological correlation, was also performed to search for biomarkers with statistical significance among the CON, DCM and ZGJTSXF groups (**Figures 4J, K**). In this study, the discriminative features of the bacterial taxa were identified with an LDA score >2.0. According to the ranked bacterial taxa, the DCM group revealed that twenty-eight communities were selectively enriched, which were Lactobacillus, Lactobacillaceae, Lactobacillales, Alistipes, Streptococcus_acidominimus, Alistipes_inops and Acidimicrobiia Microtrichales et al. After treatment, the mice of the ZGJTSXF group were enriched in thirty communities such as Ruminococcaceae_NK4A214_group, Saccharimonadia, Saccharimonadales, Candidatus_Saccharimonas, Saccharimonadaceae and Patescibacteria. Combined with the differential gut microbiota analyzed by Tukey HSD test at the genus level (**Figures 4L**), it can be concluded that Lactobacillus, Alloprevotella, Alistipes, Rikenellaceae_RC9_gut_group, Lachnospiraceae_UCG-006, Parabacteroides, Eubacterium_ventriosum_group, Odoribacter, Ruminococcaceae_UCG-013, Clostridium_sensu_stricto_1 played the most significant role in ZGJTSXF treatment.

### Diabetic cardiomyopathy-related genera regulated by ZGJTSXF

The correlations between 69 genera that changed significantly among the three groups and 18 DCM-related pathological indices were conducted by Spearman’s correlation analysis. The results were presented as a heatmap (**Supplementary Figure S3**). In general, there were 41 genera closely related to the phenotype of DCM (≥4 pathological indices were closely correlated with certain genus). Among them, Butyricicoccus, Prevotellaceae_UCG-001, Bifidobacterlum, Lachnoclostridium, Lachnospiraceae_FCSo20_group, Marvinbryantia, Blautia, Oscillibacter, UBA1819, Ruminiclostridium_5, Anaerotruncus, A2, Eubacterium_nodatum_group, GCA-900066575, Parvibacter, Ruminococcaceae_UCG-013, Eubacterium_xylanophilum_group, Desulfovibrio, Family_XIIL_UCG-001, Eubacterium_coprostanoligenes_group, Eubacterium_ventriosum_group and Candidatus_Arthromitus showed a positive correlation with FINS, HDL-C, EF%, FS% and a negative correlation with LVESv, TG, LVIDs, TC, LDL-C, blood glucose, LVIDd, LVEDv, fiber area, AUC of OGTT, HOMA-IR, cardiomyocyte apoptosis rate, Sirius red area. Alistipes, Corynebacterium_1, Escherichia-Shigella, Bifidobacterium, Klebsiella, Odoribacter, Lactobacillus, Parabacteroides, Rikenellaceae_RC9_gut_group, Alloprevotella, Clostridium_sensu_stricto_1 showed a negative correlation with FINS, HDL-C, EF%, FS% and positive correlation with with LVESv, TG, LVIDs, TC, LDL-C, blood glucose, LVIDd, LVEDv, fiber area, AUC of OGTT, HOMA-IR, cardiomyocyte apoptosis rate, Sirius red area.

### Correlations between gut microbiota and serum TMAO level

Spearman’s correlation analysis was also conducted to analyze the correlations between the 69 significant changed genera and serum TMAO level. As shown in **Supplementary Figure S4**, Rikenellaçeae_RC9_gut_group, Alloprevotella, Odoribacter, Parabacteroides, Lactobacillus, Bifidobacterium, Klebsiella, Corynebacterium_1, Alistipes and Clostridium_sensu_stricto_1 showed a positive correlation with Serum TMAO level. Eubacterium_brachy_group, Prevotellaceae_UCG-001, Marvinbryantia, Lachnospiraceae_UCG-006, Eubacterium_ventriosum_group, Ruminococcaceae_UCG-013, GCA-900066575, Desulfovibrio, Candidatus_Arthromitus, Eubacterium_coprostanoligenes_group, Lachnospiraceae_FCS020_group, Anaerotruncus, Eubacterium_xylanophilum_group, Family_XIIl_UCG-001, Ruminiclostridium_5, Eubacterium_nodatum_group, Butyricicoccus, Lachnoclostridium, A2, Oscillibacter, Blautia, Ruminococcaceae UCG-004, Roseburia, Ruminococcaceae_UCG-00, Lachnospiraceae_NK4A136_group showed a negative correlation with Serum TMAO level. Of these genera, Lactobacillus, Alloprevotella, Alistipes, Rikenellaceae_RC9_gut_group, Lachnospiraceae_UCG-006, Parabacteroides, Eubacterium_ventriosum_group, Odoribacter, Ruminococcaceae_UCG-013, Clostridium_sensu_stricto_1 could be significantly reversed by ZGJTSXF in DCM mice. Therefore, these genera might be the targets of ZGJTSXF in the treatment of DCM mice.

### ZGJTSXF administration suppressing the TMAO/PERK/FoxO1 pathway in myocardial tissues of DCM mice

**Figure 5:**
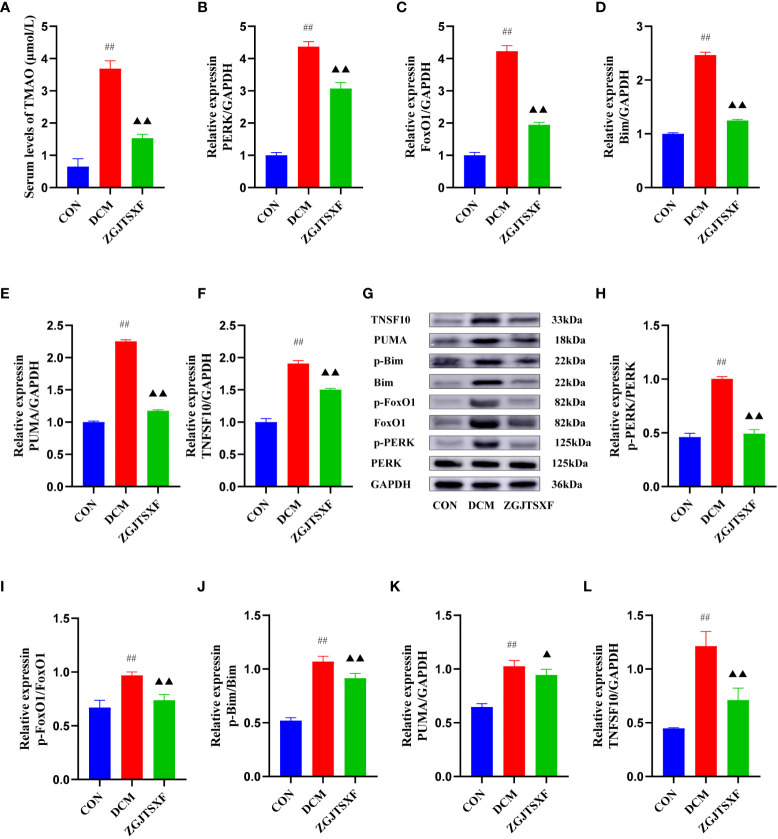
ZGJTSXF administration inhibited TMAO/PERK/FoxO1 pathway in DCM mice **(A)** The TMAO contents in serum. The mRNA levels of **(B)** PERK, **(C)** FoxO1, **(D)** Bim**(E)** PUMA**(F)** TNFSF10. **(G)** Representative images of western blot bands are shown. **(H)** The protein levels of phosphorylated PERK (p-PERK), **(I)**The protein levels of phosphorylated FoxO1 (p-FoxO1), **(J)** The protein levels of phosphorylated Bim (p-Bim), **(K)** The protein levels of PUMA, **(L)** The protein levels of TNFSF10. ^##^ *P*<0.01, compared with the CON group; ^▲▲^ *P*<0.01, ^▲^ *P*<0.05, compared with the DCM group.

In order to explored the underlying mechanism of TMAO mediated DCM. We first quantitated TMAO contents in serum *via* HPLC-MS/MS. We found that TMAO levels were higher in the DCM group than the mice in CON group. However, this was reversed by ZGJTSXF administration (*P*<0.01). Our results suggested that ZGJTSXF reduced TMAO synthesis levels in DCM mice (**Figure 5A**).

The transcription and protein levels of TMAO/PERK/FoxO1 pathway molecules and apoptosis-associated molecules (Bim, PUMA and TNFSF10) were quantitated by qPCR and western blot assays. Compared with the CON group, the DCM group had significantly increase mRNA levels of PERK, FoxO1, Bim, PUMA and TNFSF10 in the myocardial tissues (*P*<0.01). After the 4-week treatment period, compared with the DCM group, the ZGJTSXF group showed significantly reduced mRNA expression of PERK (**Figure 5B**), FoxO1 (**Figure 5C**), Bim (**Figure 5D**), PUMA (**Figure 5E**) and TNFSF10 (**Figure 5F**) in mice with DCM (*P* < 0.01). The western blot assay (**Figure 5G**) showed largely consistent expression patterns of these molecules among the groups. The ZGJTSXF treatment could significantly reduce the ratios of p-PERK/PERK (**Figure 5H**), p-FoxO1/FoxO1 (**Figure 5I**), p-Bim/Bim (**Figure 5J**), PUMA/GAPDH (**Figure 5K**), TNFSF10/GAPDH (**Figure 5L**) that were upregulated in the DCM group (*P*<0.05, 0.01). Taken together, these results demonstrated that ZGJTSXF administration inhibited apoptosis and blunted the TMAO/PERK/FoxO1 pathway in myocardial tissues of DCM mice.

## Discussion

DCM is a serious complication of diabetes. In spite of much effort, only limited progress has been made. During the present study, we demonstrated that ZGJTSXF can ameliorate the progression of DCM, and that the gut-heart axis may play an instrumental role.

Herbal medicine has been used to treat diabetes and its complications including DCM for thousands of years around the world. Most herbal medicines are taken orally and absorbed through the intestines. Normally, oral drugs go through the gastrointestinal system for metabolism (17). They interact with a large number of microorganisms in the intestine after oral medication (17, 24). It is possible to improve the body’s dysfunction and pathological conditions through herbal medicines by regulating the gut microbiota’s composition and metabolism. By participating in the metabolic transformation of herbs, the gut microbiota can also improve the bioavailability of herbal compounds (25). According to the UPLC-Q-Exactive-Orbitrap-MS analysis result, ZGJTSXF contain many bioactive compounds, including flavonoids, phenylpropanoids, organic acids, alkaloids. A small fraction of flavonoids (except flavanols) are absorbed through the small intestine as glycosides, which are naturally formed when flavonoids combine with sugar. The colonic microflora absorb glycosylated flavonoids into the body during digestion, converting them into phenolic acid and other metabolites. Microorganisms can catabolize flavonoids, changing their bioavailability and activity, regulating the colonic flora (26–36). Under the action of intestinal bacteria, phenylpropanoids undergo biotransformations such as lactone hydrolysis or demethylation (37, 38). As a prototype, organic acids are absorbed in the stomach and small intestine. Following this, they are digested further by intestinal mucosa and gut microbiota by hydrolysis by esterase. When bacteria metabolize polyphenols or carbohydrates, they produce organic acids (39, 40). It is related to Clostridium, Escherichia coli, and Lactobacillus that organic acids are produced. The formation of lactic acid regulates intestinal peristalsis and inhibits the reproduction of harmful bacteria. The biological world produces alkaloids, which are nitrogen-containing organic compounds. It is one of the most vital components of Chinese medicine because of its physiological properties. There are some alkaloids that are hydrophilic and soluble in organic solvents as well. As a result of gut microbiota action, alkaloids often consist of small molecules, ether bonds, coordination bonds, etc., which are susceptible to hydrolysis and dehydration (41–44). With all of this in mind, we could speculate that ZGJTSXF might alleviate DCM by regulating the intestinal microbiota. For this aim, we designed this study.

In high-fat and STZ-induced diabetic mice, we found ZGJTSXF administration could improve fasting blood sugar, blood lipid, fasting blood insulin and insulin resistance. Further research on echocardiography and histological analysis, we discovered that the ZGJTSXF groups can improve left ventricular systolic and diastolic functions in DCM mice. We discovered that the ZGJTSXF groups can reduce hypertrophy, vacuolar degeneration of cardiomyocytes, myocardial interstitial infiltration of inflammatory cells, capillary basement membrane thickened, and myocardial fibrosis caused by diabetic cardiomyopathy, thus improving cardiac function. Additionally, the effects of ZGJTSXF on cardiomyocyte apoptosis in DCM mice were also investigated. The results showed the ZGJTSXF groups demonstrated significantly reduced cardiomyocyte apoptosis. Although ZGJTSXF exerts many health benefits. However, it is still unclear whether gut microbiota plays a role in the occurrence of DCM.

Having a healthy gut microbiota plays an important role in maintaining normal heart function and development (8, 45–47). There has been a great deal of interest in recent years regarding the presence of gut microbiota and specific gut microbiota-dependent pathways as well as downstream metabolites in cardiovascular disease and metabolic disorders (8, 45). In the present study, we used 16S gene rRNA sequencing to identify changes in gut microbial diversity and composition following ZGJTSXF treatment. A recent study found that metabolic diseases are associated with decreased gut microbial diversity (48). Our result is consistent with recent studies, we also observed significant decreases in Sobs, Chao1, ACE, and Shannon indexes in DCM model mice. It was, however, reversed by ZGJTSXF. Based on UPGMA and PCoA, there was a clear cluster separation between ZGJTSXF mice and DCM mice. The ZGJTSXF group had a smaller distance from the CON group, indicating that the ZGJTSXF treatment significantly normalized the biological community structure.

The results of LEfSe analysis and environmental factor correlation analysis showed that Lactobacillus, Alloprevotella, Alistipes, Rikenellaceae_RC9_gut_group, Lachnospiraceae_UCG-006, Parabacteroides, Eubacterium_ventriosum_group, Odoribacter, Ruminococcaceae_UCG-013, Clostridium_sensu_stricto_1 played important roles in DCM. Lactobacillus genus represents a common probiotic, but this protective effect may depend on the presence of other intestinal bacteria and work together in a complementary manner (49). Most other studies have reported that Lactobacillus was found in higher abundance in control groups than in diabetic groups (50, 51). But here, the abundance of Lactobacillus being significantly higher in the DCM group than in the CON group. Other studies have produced similar results on the abundance of Lactobacillus in diabetic patients (52, 53). Research has shown that increased Lactobacilli levels were positively correlated with fasting glucose, glycosylated hemoglobin, and a long-term measure of blood glucose control (54). Thus, increased Lactobacillus in the intestines could be a consequence of increased glucose levels in diabetic patients. On the other hand, lactobacilli also have the potential to cause infections, but this is considered rare. Lactobacilli-induced infections are more common in immunocompromised and diabetic patients, but sometimes also in subjects without underlying diseases or risk factors (55). Here, we also found that Alloprevotella genus was significantly increased in DCM group. In addition, we found that Alloprevotella were positively correlated with levels of LVESv, TG, LVIDs, TC, LDL-C, blood glucose, LVIDd, LVEDv, fiber area, AUC of OGTT, HOMA-IR, cardiomyocyte apoptosis rate, blood glucose, Sirius red area but negatively correlated with FINS, HDL-C, EF%, FS%, indicating that Alloprevotella genus may contribute to DCM. The specific function of the Rikenellaceae_RC9_gut_group is currently unknown, but the Rikenellaceae_RC9_gut_group is closely related to members of the genus Alistipes, and Alistipes can produce TMAO (56). It was reported that Lachnospiraceae_UCG-006 were potentially related to obesity and inflammation. Increase the the abundance of Lachnospiraceae_UCG-006 was reported to improve insulin resistance and reduce inflammation (57). Parabacteroides is also known to be associated with diet-induced obesity. Lecomte et a. reported that Parabacteroides is one of the major succinate producers in the gut, and is also associated with obesity (58). Eubacterium_ventriosum_group belongs to Lachnospiraceae. Dang et al. showed that decrease of Eubacterium_ventriosum_group was associated with enhanced systemic inflammation (59). In a previous study, Odoribacter was reported to be highly abundant in hypercholesterolemic subjects and the isobutyric acid proportion was positively associated with Odoribacter abundance (60). In addition, Odoribacter was also reported to exhibit higher abundance in diabetic mice and cause some health problems such as abdominal inflammation (61). Clostridium_sensu_stricto_1, a genus belonging to butyrate-producing Clostridia bacteria, the proliferation of Clostridium species in the colon may produce toxins and lead to intestinal epithelial damage (62).

Furthermore, the correlation analysis between the differential intestinal microbiota and serum TMAO level showed that serum TMAO level were positively associated with Lactobacillus, Alloprevotella, Alistipes, Parabacteroides, Odoribacter and Clostridium_sensu_stricto_1 but negatively with Lachnospiraceae_UCG-006, Eubacterium_ventriosum_group, Ruminococcaceae_UCG-013. Interestingly, we found that ZGJTSXF treatment reduced the abundance of gut bacteria positively correlated with TMAO and increased the abundance of gut bacteria negatively correlated with TMAO. TMAO is an oxidation product of trimethylamine (TMA), which is produced by gut microbiota from dietary choline and phosphatidylcholine. After absorption in the gut, TMA reaches the liver where it is converted to TMAO by hepatic flavin-containing monooxygenases(FMO3) (63, 64). In recent years, TMAO have been extensively studied due to their potential cardiovascular risks (10–12). According to the current evidence, ZGJTSXF is believed to reduce DCM by regulating the intestinal microbiota. But it is still elusive whether ZGJTSXF improve DCM Mice by alleviation gut - heart axis.

There have been several studies demonstrating the link between high circulating TMAO levels and a number of diseases, including metabolic syndrome (65, 66), insulin resistance (66), obesity (67), and nonalcoholic fatty liver disease (68). Interestingly, when we analyzed the gut microbiota, we found an altered increased abundance in the TMAO-producing gut microbiota. Therefore, we hypothesized that the gut microbiota communicates with the host through the small molecule metabolite-TMAO. Thus, we next examined the metabolites of the gut microbiota, TMAO, and how TMAO communicates with the heart, providing important evidence to support the gut-heart axis. We found that the TMAO concentration in the model group was higher than that in the control group; while the TMAO concentration in the ZGJTSXF group were significantly downregulated. PERK is an endoplasmic reticulum (ER) stress sensor. When TMAO is absorbed at physiologically relevant concentrations, it activates the transcription factor PERK, which in turn induces FoxO1 (14). Studies have shown that excessive activation of cardiac FoxO1 causes DCM and heart failure *via* insulin receptor substrate downregulation (69). Cardiomyocyte apoptosis is a crucial factor leading to myocardial dysfunction (70). The activation of FoxO1 has recently been demonstrated to modulate the expression of genes involved in apoptosis, including Bim, PUMA, TNFSF10. Previous data have suggested that Bim, PUMA, TNFSF10 is a downstream target of FoxO1 transcription factors. Recently, Bim, PUMA, TNFSF10 has been shown to be mediated by FoxO1-induced apoptosis (71–73). Our results are consistent with the above findings. In the DCM state, gut microbiota dysbiosis leads to the increase of TMAO levels in the circulating. TMAO binds and induces phosphorylation of PERK in the cardiomyocytes and then PERK induced FoxO1, which promoted cardiac fibrosis, dysfunction and cardiomyocyte apoptosis. In this research, the high expression levels of PERK, FoxO1 in DCM mice were reversed after ZGJTSXF treatment. The results showed that the preventative effect of ZGJTSXF alleviated DCM were due to blunte the expression of key genes and proteins in the TMAO/PERK/FoxO1 signaling pathway which is associated with its modulation on gut microbiota imbalance.

## Conclusion

In conclusion, the present study showed that ZGJTSXF could ameliorate DCM. The therapeutic effect of ZGJTSXF on DCM mice might be mediated by changes in the gut microbiotaand its metabolite levels. The findings suggested that ZGJTSXF was a promising complementary option for DCM. The efficacy and safety of ZGJTSXF are worth investigating in the clinic in the future.

## Data availability statement

The data presented in the study are deposited in the SRA database, accession number PRJNA924008.

## Ethics statement

The animal study was reviewed and approved by EthicsCommittee of Hunan University of Traditional Chinese Medicine (Hunan, China).

## Author contributions

Y-LH, QX, and RY designed the experiment. Y-LH, and QX performed the experiment. Y-LH and YW analyzed the data and wrote the manuscript. Y-LH, QX, J-JZ, YW and RY revised the manuscript. All authors contributed to the article and approved the submitted version.
