# Supplementary material for: Zuogui Jiangtang Shuxin formula Ameliorates diabetic cardiomyopathy mice via modulating gut-heart axis
Source: Front Endocrinol (Lausanne). 2023 Feb 9;14:1106812. doi: 10.3389/fendo.2023.1106812 (PMC9948445; doi:10.3389/fendo.2023.1106812)
Supplement: Supplementary file 1 [file DataSheet_1.pdf]

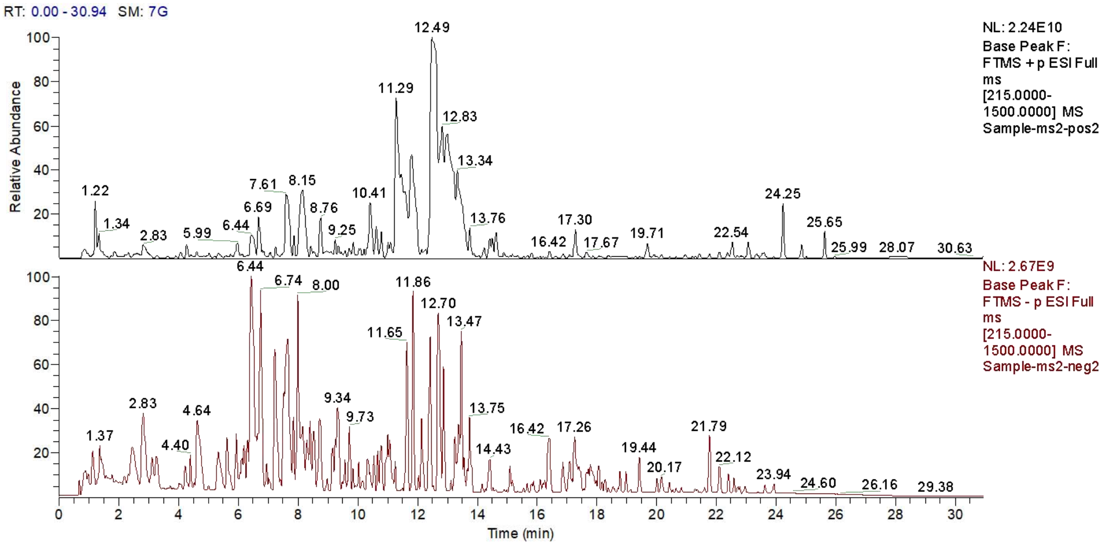

Supplementary Figure S1 ZGJTSXF UHPLC-Q-Orbitrap positive and negative ion modes Total ion flow diagram under

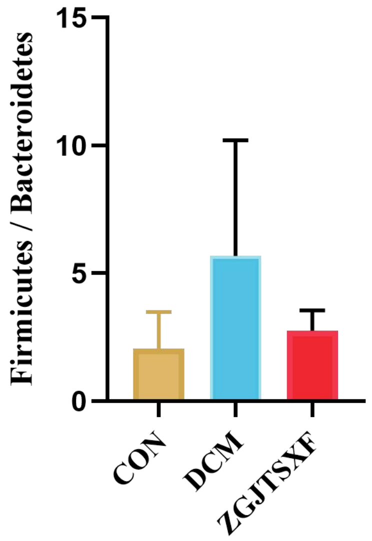

Supplementary Figure S2 ZGJTSXF reduced the ratio of Firmicutes to Bacteroidetes

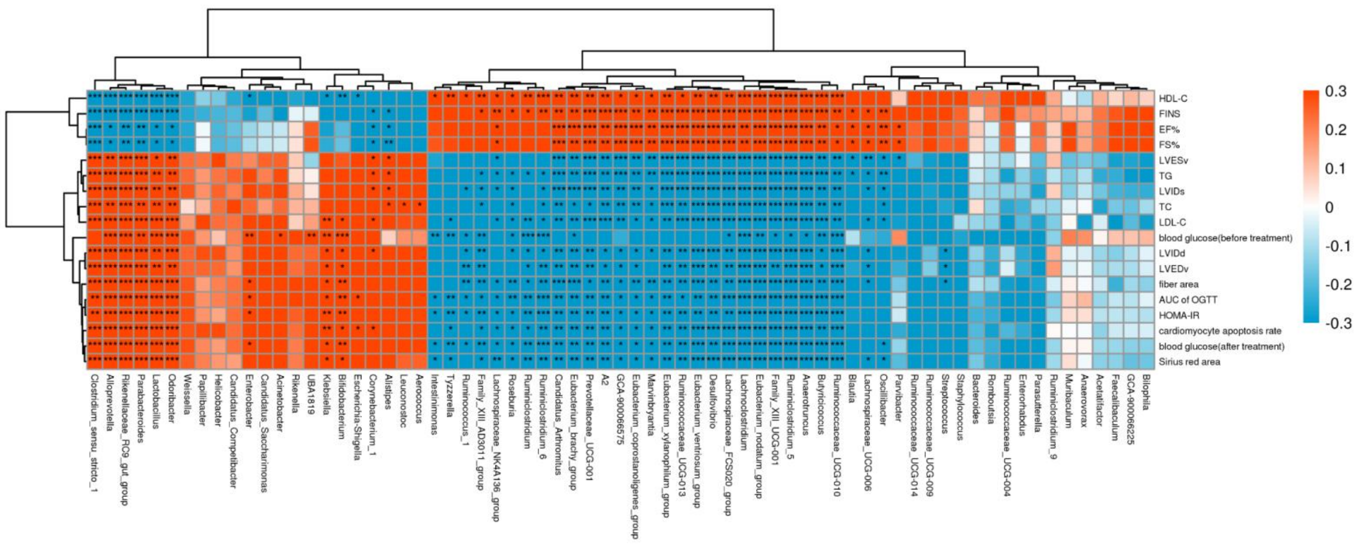

Supplementary Figure S3 The correlations between 69 genera and 18 DCM-related pathological indices by Spearman's correlation analysis

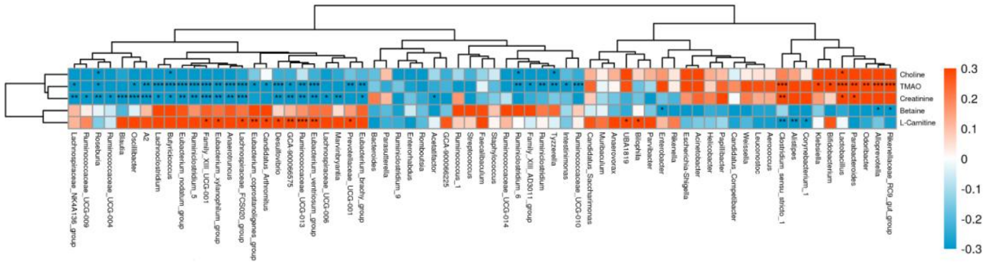

Supplementary Figure S4 69 significant changed genera and Serum TMAO level by Spearman's correlation analysis

Supplementary Table S1 tthe ZGJTSXF of Chemical composition mass spectrometry information

No.	RT [min]	Precursor ions	Adduct	Formula	Identification	MW	Diff. ppm	ns/ms ragment ions					Compound Class	Origin plant
1	0.718	259.0232544	M-H	$C_6H_{13}O_9P$	D-Glucose 6-phosphate	260.03053	3.13	96.96955	78.95904	259.02252	96.96045	138.98036	oligoses	Crataegus pinnatifida Bge
2	0.754	131.0462341	M-H	$C_4H_8N_2O_3$	Asparagine	132.05351	0.14	114.01959	113.03566	70.02981	131.04617	95.02505	acids/small peptides	Astragalus
3	0.757	503.1621094	M-H	$C_{18}H_{32}O_{16}$	D-Raffinose	504.16934	0.61	179.05612	59.01383	383.11972	89.02441	71.01384	oligoses	jinseng/Bunge. Crataegus
4	0.801	177.0988617	M+H	$C_5H_{12}N_4O_3$	L-Canavanine	176.09157	3.6	76.05056	160.07166	118.04987	72.04445	177.09865	acids/small peptides	
5	0.931	175.1191101	M+H	$C_6H_{14}N_4O_2$	DL-Arginine	174.11185	1.01	70.06522	175.11899	60.05593	116.07062	130.09756	acids/small peptides	Astragalus
6	0.959	244.0930176	M+H	$C_9H_{13}N_3O_5$	Cytidine	243.08572	0.83	112.05056	95.02399	69.04485	94.04008	68.34165	nucleosides	Bunge
7	0.981	291.130127	M+H	$C_{10}H_{18}N_4O_6$	rgininosuccini acid	290.12285	0.75	291.13	70.06526	175.11908	60.05594	71.04922	acids/small peptides	
8	1.034	323.0285339	M-H	$C_9H_{13}N_2O_9P$	Uridine monophosphat e (UMP)	324.03581	-0.17	211.00148	78.95907	111.02001	323.0289	96.96961	nucleosides	Bunge
9	1.049	152.070755	M+H	$C_8H_9NO_2$	-Phenylglycin	151.06348	0.99	152.0706	106.06515	93.05733	134.06018	153.07494	acids/small peptides	
10	1.074	330.0586853	M+H	$C_{10}H_{12}N_5O_6P$	Adenosine 3'5'-cyclic monophosphat e	329.05141	-3.38	136.06184	330.05981	98.98419	69.03362	119.03532	nucleosides	Bunge
11	1.096	344.0400696	M-H	$C_{10}H_{12}N_5O_7P$	Guanosine cyclic monophosphat e	345.0475	0.2	150.04204	133.01555	344.04007	108.02032	107.03622	nucleosides	Bunge
12	1.101	191.0198364	M-H	$C6H8O7$	Citric acid	192.02711	0.58	111.00878	87.00878	85.02955	191.01991	129.01939	organic acids	pinnatifida Bge/Panax
13	1.104	154.0976257	M+H	$C_7H_{11}N_3O$	-Acetylhistam ne	153.09035	0.9	154.0975	70.06525	94.06518	112.07574	154.04976	acids/small peptides	
14	1.104	187.1078949	M+H	$C_8H_{14}N_2O_3$	-Alanyl-L-prol ne	186.1006	0.86	116.07066	187.10762	70.06525	169.09706	72.08086	acids/small peptides	
15	1.12	113.0346832	M+H	$C_4H_4N_2O_2$	Uracil	112.02738	0.93	113.03469	70.02885	96.00801	113.02348	113.0598	nucleosides	Bunge
16	1.126	173.0091553	M-H	$C_6H_6O_6$	trans-Aconitic acid	174.01643	-0.05	85.0295	129.01935	111.00871	173.00954	86.03286	organic acids	Cornus officinalis Sieb

No.	RT [min]	Precursor ions	Adduct	Formula	Identification	MW	Diff. ppm	ms/ms fragment ions					Compound Class	Origin plant
17	1.139	190.0712128	M+H	$C_7 H_{11} N O_5$	-Acetyl-L-glu amic acid	189.06392	1.06	130.04996	84.04443	102.05501	148.06041	61.03994	acids/small peptides	
18	1.149	156.0770721	M+H	$C_6 H_9 N_3 O_2$	L-Histidine	155.06976	1.81	110.07133	156.0768	156.10194	138.05493	86.06006	acids/small peptides	Astragalus
19	1.176	180.1021271	M+H	$C_{10} H_{13} N O_2$	Salsolinol	179.09485	1.24	180.10185	163.07527	145.0648	85.02843	117.0699	alkaloids	optis chinensi Franch
20	1.222	233.149826	M+H	$C_{10} H_{20} N_2 O_4$	-Isoleucyl-L-t reonine	232.14255	1.04	86.09644	69.07001	233.14946	120.06564	215.13908	acids/small peptides	
21	1.223	268.1042786	M+H	$C_{10} H_{13} N_5 O_4$	Adenosine	267.097	0.93	136.06175	268.10388	119.0352	137.04572	94.03992	nucleosides	Bunge
22	1.232	247.1291656	M+H	$C_{10} H_{18} N_2 O_5$	-Isoleucyl-L-a partic acid	246.12183	1.05	86.09642	69.06999	247.12881	72.08086	56.04981	acids/small peptides	
23	1.24	252.109314	M+H	$C_{10} H_{13} N_5 O_3$	2'-Deoxyaden sine	251.10204	0.79	136.06189	252.10757	119.03526	117.05479	137.04585	nucleosides	Bunge
24	1.242	152.0567932	M+H	$C_5 H_5 N_5 O$	Guanine	151.04952	0.71	152.05682	152.07051	153.04077	135.03014	110.03485	nucleosides	Bunge
25	1.243	117.0193329	M-H	$C_4 H_6 O_4$	Succinic acid	118.02661	0.01	73.02949	117.0193	99.00874	74.03288	118.0227	organic acids	jinseng/Bunge Crataegus
26	1.292	261.1447144	M+H	$C_{11} H_{20} N_2 O_5$	Ile-Glu	260.13743	0.79	86.09648	261.14478	69.07004	148.06053	132.10201	acids/small peptides	
27	1.33	229.154892	M+H	$C_{11} H_{20} N_2 O_3$	Prolylleucine	228.14758	0.83	229.15471	70.06523	98.06007	142.08632	86.06003	acids/small peptides	
28	1.353	103.0036469	M-H	$C_3 H_4 O_4$	Malonic acid	104.01092	-0.34	59.01383	103.0037	103.04001	73.02949	57.03455	organic acids	Crataegus pinnatifida Bge
29	1.373	116.0707169	M+H	$C_5 H_9 N O_2$	D-(+)-Proline	115.06345	1.07	70.06521	116.0706	71.06847	71.04916	68.04954	acids/small peptides	Astragalus
30	1.443	169.0142822	M-H	$C_7 H_6 O_5$	Gallic acid	170.02155	0.14	125.02444	169.01434	69.03465	97.02953	81.03461	organic acids	Cornus officinalis Sieb
31	1.583	483.078064	M-H	$C_{20} H_{20} O_{14}$	Hamamelitanni n	484.08539	0.18	169.01424	483.07828	125.02438	331.06729	107.01382	phenols	Cornus officinalis Sieb
32	1.592	298.1148682	M+H	$C_{11} H_{15} N_5 O_5$	7-Methylguan sine	297.10759	0.92	166.07243	149.04585	167.05634	153.04074	298.1373	nucleosides	Bunge
33	1.612	105.0192871	M-H	$C_3 H_6 O_4$	DL-Glyceric acid	106.02656	-0.42	105.01925	72.99309	75.00871	59.01382	69.92811	organic acids	Crataegus pinnatifida Bge
34	1.75	282.119873	M+H	$C_{11} H_{15} N_5 O_4$	2'-O-Methylad enosine	281.11227	-0.48	136.06183	282.11948	119.03525	69.03362	137.04579	nucleosides	Bunge
35	1.758	361.1141357	M-H	$C_{15} H_{22} O_{10}$	Catalpol	362.12111	1.74	169.01424	125.02438	271.0462	361.07776	59.01383	iridoids	Bunge

No.	RT [min]	Precursor ions	Adduct	Formula	Identification	MW	Diff. ppm	ms/ms fragment ions					Compound Class	Origin plant
36	1.974	298.1148682	M+H	$C_{11}H_{15}N_5O_5$	2'-O-Methylguanosine	297.10756	0.8	152.05681	153.04082	135.03024	101.05983	110.035	nucleosides	Bunge
37	2.11	217.1547699	M+H	$C_{10}H_{20}N_2O_3$	Valylvaline	216.14749	0.43	72.08083	217.15524	55.05461	118.08625	217.12714	acids/small peptides	
38	2.137	341.1087952	M-H	$C_{12}H_{22}O_{11}$	α,α-Trehalose	342.11607	-0.41	89.02444	59.01384	71.01385	161.04564	179.05621	oligoses	pinnatifida
39	2.138	303.1453552	M+H	$C_{15}H_{18}N_4O_3$	L-Histidyl-L-phenylalanine	302.13808	0.62	110.07129	303.1442	83.06035	93.04475	285.13437	acids/small peptides	
40	2.172	141.0193481	M-H	$C_6H_6O_4$	cis,cis-Muconic acid	142.02662	0.09	97.02949	69.0346	141.0195	67.01896	95.01386	organic acids	pinnatifida
41	2.24	633.0737305	M-H	$C_{27}H_{22}O_{18}$	Corilagin	634.08101	0.62	300.99905	633.07361	275.02005	249.04053	231.02974	phenols	officinalis
42	2.254	197.00914	M-H	$C_8H_6O_6$	4,5-Dihydroxyphthalic acid	198.01642	-0.11	153.01933	109.02944	197.0092	151.06117	197.04681	phenols	Bunge
43	2.277	731.2258301	M+FA-H	$C_{27}H_{42}O_{20}$	Rehmannioside D	686.22747	0.77	179.05615	263.07733	71.01385	59.01382	89.02444	iridoids	Bunge
44	2.356	166.0864105	M+H	$C_9H_{11}NO_2$	L-Phenylalanine	165.07913	0.89	120.0808	103.05421	166.08623	93.0699	131.04916	acids/small peptides	
45	2.444	127.0390625	M+H	$C_6H_6O_3$	5-(Hydroxymethyl)-2-furaldehyde	126.03179	0.8	109.02848	81.03355	127.03903	53.03897	110.03184	/	Bunge
46	2.627	289.09198	M+H	$C_{12}H_{16}O_8$	Dianthoside	288.0847	0.65	127.03902	85.02843	69.03362	109.02843	81.03356	saponins	Ophiopogon japonicus
47	2.712	167.03508	M-H	$C_8H_8O_4$	Homogentisic acid	168.04236	0.6	167.03488	123.04512	68.99574	139.04001	93.03451	phenols	Bunge
48	2.764	315.0723267	M-H	$C_{13}H_{16}O_9$	Gentisic acid 5-O-glucoside	316.0796	0.54	315.07248	108.02165	152.01147	109.0294	153.01926	phenols	Pueraria lobata
49	2.852	197.0455017	M-H	$C_9H_{10}O_5$	Danshensu	198.05273	-0.46	72.9931	135.0452	123.04519	179.03505	197.04561		miltiorrhiza
50	3.04	158.0814209	M+H	$C_7H_{11}NO_3$	AC-D-PRO-OH	157.07414	1.57	70.06519	112.07566	116.07056	158.08119	140.07047	acids/small peptides	
51	3.12	483.0779724	M-H	$C_{20}H_{20}O_{14}$	1,6-Bis-O-(3,4,5-trihydroxybenzoyl)hexopyranose	484.08533	0.06	169.01421	483.07822	125.02436	331.0668	313.0567	phenols	Pueraria lobata
52	3.234	295.1287842	M+H	$C_{14}H_{18}N_2O_5$	Phe-glu	294.12155	-0.06	120.08084	295.12909	103.05425	148.06049	84.04443	acids/small peptides	
53	3.239	153.0193481	M-H	$C_7H_6O_4$	Gentisic acid	154.02663	0.11	109.02941	153.01929	108.02169	110.03285	81.03459	phenols	Pueraria lobata
54	3.284	219.13414	M+H	$C_9H_{18}N_2O_4$	D-Seryl-L-leucine	218.12684	0.82	60.04469	86.09643	132.10196	219.13385	173.12854	acids/small peptides	

No.	RT [min]	Precursor ions	Adduct	Formula	Identification	MW	Diff. ppm	ms/ms fragment ions					Compound Class	Origin plant
55	3.319	189.1235352	M+H	$C_8H_{16}N_2O_3$	Gly-Ile	188.11624	0.81	86.09643	143.11795	132.10197	75.05536	189.07396	acids/small peptides	
56	3.396	169.0496674	M+H	$C_8H_8O_4$	Isovanillic acid	168.04239	0.79	111.04411	125.05978	93.03354	65.03879	169.04955	phenols	Panax ginseng
57	3.406	329.0878601	M-H	$C_{14}H_{18}O_9$	Vanillic acid 4-β -D-glucoside	330.09511	0.08	167.03496	108.02166	152.01146	123.04519	329.08749	phenols	miltiorrhiza
58	3.474	246.1450348	M+H	$C_{10}H_{19}N_3O_4$	Asn-Leu	245.13773	0.72	229.11853	212.09184	110.02376	141.10233	87.05537	acids/small peptides	
59	3.571	437.2145691	M+H	$C_{19}H_{28}N_6O_6$	asp-phe-arg	436.20728	0.56	120.0808	437.21463	70.06522	322.18738	175.11896	acids/small peptides	
60	3.741	153.0557556	M-H	$C_8H_{10}O_3$	,4-Dihydroxyphenylethar ol	154.06303	0.26	109.02947	123.0452	153.01944	153.05569	108.02177	phenols	miltiorrhiza
61	3.945	189.1235352	M+H	C8 $H_{16}N_2O_3$	Glycyl-L-leucine	188.11624	0.79	86.09644	132.10197	143.11797	190.08615	189.10225	acids/small peptides	
62	4.06	497.0923157	M+H	$C_{21}H_{20}O_{14}$	4,5-di-O-galloylquinic acid	496.08504	-0.54	153.01817	139.03893	79.01781	143.03386	153.02892	phenols	miltiorrhiza
63	4.095	312.1303406	M+H	$C_{12}H_{17}N_5O_5$	N2-Dimethylguanosine	311.12307	0.33	180.08795	110.03492	153.04073	84.04442	69.03363	nucleosides	Bunge
64	4.139	373.1138916	M-H	$C_{16}H_{22}O_{10}$	geniposidic acid	374.12135	0.14	123.04517	149.06073	211.06131	59.01384	167.07137	iridoids	Bunge
65	4.209	113.0598145	M+H	$C_6H_8O_2$	Sorbic acid	112.05254	0.97	113.05974	67.0544	95.04918	71.04924	85.06485	organic acids	Crataegus pinnatifida Bge
66	4.216	167.0704651	M+H	$C_9H_{10}O_3$	Apocynin	166.06319	1.17	149.0598	121.06487	93.06995	167.07027	91.05428	phenols	Astragalus
67	4.299	421.1343384	M+H	$C_{17}H_{24}O_{12}$	'-O-beta-D-apiofuranos l-beta-D-glucopyranosic	420.12706	0.68	127.039029	289.09167	85.02841	69.03363	68.84815	oligoses	Crataegus pinnatifida Bge
68	4.42	261.1447449	M+H	$C_{11}H_{20}N_2O_5$	Glu-Leu	260.13744	0.83	84.04443	243.13405	86.09641	197.12854	132.10196	acids/small peptides	
69	4.522	279.1343384	M+H	$C_{14}H_{18}N_2O_4$	Tyrosyl-L-proline	278.12705	1.4	136.07579	116.07065	91.05427	70.06524	279.13397	acids/small peptides	
70	4.583	183.0298004	M-H	$C_8H_8O_5$	,4-Dihydroxy-5-methox benzoic acid	184.03719	0.1	168.00638	124.01653	183.02985	139.0401	183.07741	phenols	Astragalus
71	4.658	231.1705322	M+H	$C_{11}H_{22}N_2O_3$	Ile-val	230.16326	0.93	86.09643	69.07001	231.17018	231.07611	84.08085	acids/small peptides	
72	4.742	355.102417	M+H	$C_{16}H_{18}O_9$	Neochlorogenic acid	354.09508	0	163.03902	135.0441	89.03861	117.03355	145.02846	phenylpropanoids	Crataegus pinnatifida Bge
73	4.873	146.117691	M+H	$C_7H_{15}NO_2$	Methyl L-isoleucinate	145.11041	0.94	86.0964	69.07001	146.11755	72.08071	100.04741	acids/small peptides	
74	4.984	231.1705322	M+H	$C_{11}H_{22}N_2O_3$	D-Valyl-D-alloisoleucine	230.16325	0.89	72.08085	55.05463	231.17073	86.09645	132.10199	acids/small peptides	

No.	RT [min]	Precursor ions	Adduct	Formula	Identification	MW	Diff. ppm	ms/ms fragment ions					Compound Class	Origin plant
75	5.008	137.024353	M-H	$C_7 H_6 O_3$	Salicylic acid	138.03164	-0.38	137.0244	136.0166	138.02782	109.02963	108.02172	phenols	Astragalus
76	5.077	158.0822449	M-H	$C_7 H_{13} N O_3$	N-Acetyl-DL-norvaline	159.08957	0.19	116.0717	158.08226	114.09244	159.02986	73.02948	acids/small peptides	
77	5.081	371.0983582	M+FA-H	$C_{15} H_{18} O_8$	p-Coumaric acid glucoside	326.10018	0.04	191.05615	163.0401	119.05019	85.02948	93.03449	phenylpropanoids	Crataegus pinnatifida Bge
78	5.122	231.1705322	M+H	$C_{11} H_{22} N_2 O_3$	Leu-Val	230.16325	0.88	86.09644	231.17049	72.08087	185.16499	118.08624	acids/small peptides	
79	5.334	298.0969849	M+H	$C_{11} H_{15} N_5 O_3 S$	5'-S-Methyl-5'-thioadenosine	297.08971	0.5	136.06181	298.09689	61.01092	75.02634	119.03523	nucleosides	Bunge
80	5.483	295.1654358	M+H	$C_{15} H_{22} N_2 O_4$	L-leucyl-D-tyrosine	294.15816	0.68	86.09642	69.07001	295.16544	182.0813	136.07576	acids/small peptides	
81	5.497	281.1134644	M+H	$C_{13} H_{16} N_2 O_5$	α-Aspartylphenylalanine	280.10616	0.85	120.08083	166.08629	88.03934	91.05424	103.05421	acids/small peptides	
82	5.571	121.0649261	M+H	$C_8 H_8 O$	Acetophenone	120.05765	1.12	121.06491	93.06996	103.05427	91.0543	95.0492	acids/small peptides	
83	5.581	299.113739	M-H	$C_{14} H_{20} O7$	Salidroside	300.12101	0.36	59.01384	71.01385	89.02445	119.05005	119.03504	phenols	Astragalus
84	5.612	263.1425781	M+H	$C_{11} H_{22} N_2 O_3 S$	ile-met	262.1353	0.72	86.09645	263.14441	69.07003	150.05833	263.10248	acids/small peptides	
85	5.633	229.1549225	M+H	$C_{11} H_{20} N_2 O_3$	Leucylproline	228.14764	1.1	116.07057	86.09637	70.06519	229.15466	68.88325	acids/small peptides	
86	5.715	261.1446228	M+H	$C_{11} H_{20} N_2 O_5$	Glu-Ile	260.13733	0.43	84.04442	86.09641	132.10196	261.14468	198.11235	acids/small peptides	
87	5.894	272.1282043	M+H	$C_{16} H_{17} N O_3$	Demethylcoclaurine	271.12093	0.31	107.04918	272.12836	255.10173	161.05974	115.05427	alkaloids	Coptis chinensis Franch
88	5.91	231.1705322	M+H	$C_{11} H_{22} N_2 O_3$	Val-Leu	230.16325	0.88	72.08083	231.17039	55.05462	86.09641	132.10194	acids/small peptides	
89	5.932	295.1652527	M+H	$C_{15} H_{22} N_2 O_4$	L-Isoleucyl-D-tyrosine	294.15798	0.08	86.09646	295.1655	182.08138	136.07584	165.05482	acids/small peptides	
90	5.954	261.1233521	M+H	$C_{14} H_{16} N_2 O_3$	cyclo(tyrosyl-prolyl)	260.11608	-0.06	261.12311	70.06522	107.04913	136.07573	188.07056	acids/small peptides	
91	5.983	247.1441803	M+H	$C_{14} H_{18} N_2 O_2$	lenticin	246.13692	0.37	188.0706	146.06003	60.08107	118.06512	144.08083	alkaloids	Coptis chinensis Franch
92	6.015	337.0930176	M-H	$C_{16} H_{18} O_8$	3-O-p-Coumaroylquinic acid	338.10031	0.43	163.0401	119.0502	191.05614	337.09348	173.04591	phenylpropanoids	Crataegus pinnatifida Bge

No.	RT [min]	Precursor ions	Adduct	Formula	Identification	MW	Diff. ppm	ms/ms fragment ions					Compound Class	Origin plant
93	6.021	327.1087341	M-H	$C_{15}H_{20}O_8$	Androsin	328.11598	0.48	147.04512	59.01382	71.01382	89.02442	101.02435	phenols	Astragalus
94	6.054	227.0913239	M+H	$C_{11}H_{14}O_5$	Sarracenin	226.08405	-0.34	139.03905	195.06528	81.03355	141.05469	95.0492	iridoids	Bunge
95	6.149	375.1295776	M-H	$C_{16}H_{24}O_{10}$	Loganic acid	376.13697	0.05	213.077	375.12973	69.03458	59.01385	113.02444	iridoids	Bunge
96	6.214	311.0408936	M-H	$C_{13}H_{12}O_9$	Caftaric acid	312.04817	0.14	179.03508	135.04523	149.00916	87.00876	59.01385	phenylpropano ds	Crataegus pinnatifida Bge
97	6.277	289.072113	M-H	$C_{15}H_{14}O_6$	Epicatechin	290.07923	0.66	289.07187	109.02943	245.08182	123.04517	125.02441	flavonoids	Crataegus pinnatifida Bge
98	6.347	497.0923157	M+H	$C_{21}H_{20}O_{14}$	3,5-di-O-galloylquinic acid	496.08504	-0.54	153.01834	79.0179	143.03401	153.02896	125.02345	phenols	Salvia miltiorrhiza Bunge
99	6.401	353.0878601	M-H	$C_{16}H_{18}O_9$	Chlorogenic acid	354.09516	0.22	191.05621	85.02952	93.03442	353.08652	127.04016	phenylpropano ds	Crataegus pinnatifida Bge
100	6.436	165.0193176	M-H	$C_8H_6O_4$	3-formylsalicylic acid	166.02661	0	121.02947	165.01929	122.03294	93.03452	77.03967	phenols	Salvia miltiorrhiza Bunge
101	6.467	139.0389557	M+H	$C_7H_6O_3$	3,4-Dihydroxybenzaldehyde	138.03168	-0.11	139.03899	111.04408	83.04913	79.01785	68.02577	phenols	Salvia miltiorrhiza Bunge
102	6.47	405.1405334	M-H	$C_{17}H_{26}O_{11}$	Morroniside	406.14751	-0.01	101.02437	155.03502	141.05577	243.08743	68.99821	iridoids	Cornus officinalis Sieb
103	6.486	252.0878296	M-H	$C_{12}H_{15}NO_5$	N-Acetylvanilalanine	253.09511	0.33	252.08775	210.07726	193.07442	122.03735	134.03737	acids/small peptides	
104	6.491	265.1546326	M+H	$C_{14}H_{20}N_2O_3$	Phe-Val	264.14735	-0.15	120.08086	103.05427	265.15506	72.08083	93.06991	acids/small peptides	
105	6.55	497.0923157	M+H	$C_{21}H_{20}O_{14}$	3,4-di-O-galloylquinic acid	496.08504	-0.54	153.01833	79.01788	143.03406	153.02913	125.0235	phenols	Salvia miltiorrhiza Bunge
106	6.552	263.142395	M+H	$C_{11}H_{22}N_2O_3S$	Met-Ile	262.13512	0.02	104.05283	56.04984	61.01091	263.14227	86.09644	acids/small peptides	
107	6.637	121.0295258	M-H	$C_7H_6O_2$	Benzoic acid	122.03685	0.61	121.02946	122.03284	108.02167	93.03454	122.02454	/	Bunge
108	6.689	433.112854	M+H	$C_{21}H_{20}O_{10}$	Pueraria glycoside	432.10557	-0.17	433.11295	313.07065	283.06009	415.10242	255.06516	flavonoids	Pueraria lobata

No.	RT [min]	Precursor ions	Adduct	Formula	Identification	MW	Diff. ppm	ms/ms fragment ions					Compound Class	Origin plant
109	6.722	325.0929565	M-H	$C_{15}H_{18}O_8$	Melilotoside	326.10023	0.2	119.0502	163.04015	325.09851	145.05058	89.02451	phenylpropanoids	Crataegus pinnatifida Bge
110	6.731	245.1859131	M+H	$C_{12}H_{24}N_2O_3$	Leu-Leu	244.17864	-0.23	86.0964	245.18591	69.06999	199.18036	132.10176	acids/small peptides	
111	6.806	159.0662384	M-H	$C_7H_{12}O_4$	Pimelic acid	160.07353	-0.19	97.06582	159.06619	115.07639	95.05022	159.02994	organic acids	Crataegus pinnatifida Bge
112	6.818	367.1033936	M-H	$C_{17}H_{20}O_9$	5-O-feruloyl-D-quinic acid	368.11071	-0.05	193.05063	134.03732	367.1033	117.03461	149.06068	phenylpropanoids	Crataegus pinnatifida Bge
113	6.871	353.0878906	M-H	$C_{16}H_{18}O_9$	Cryptochlorogenic acid	354.09517	0.26	173.04555	135.04521	179.03506	191.05617	353.08875	phenylpropanoids	Crataegus pinnatifida Bge
114	6.939	276.1345215	M+H	$C_{14}H_{17}N_3O_3$	D-Alanyl-D-tryptophan	275.12724	0.9	188.0706	205.09718	146.06004	276.13458	118.06515	acids/small peptides	
115	6.968	389.1090393	M-H	$C_{16}H_{22}O_{11}$	Secologanoside	390.11636	0.37	69.0346	59.01385	121.06586	389.10947	71.01389	iridoids	Cornus officinalis Sieb
116	7.052	565.1550293	M+H	$C_{26}H_{28}O_{14}$	Isovitexin 2''-O-xyloside	564.14785	-0.1	433.11295	565.15509	313.07062	283.06018	255.06511	flavonoids	Pueraria lobata
117	7.159	295.1654053	M+H	$C_{15}H_{22}N_2O_4$	Tyr-Leu	294.15812	0.56	136.07576	91.05424	119.04921	295.16525	86.09641	acids/small peptides	
118	7.246	577.135376	M-H	$C_{30}H_{26}O_{12}$	Procyanidin B2	578.14234	-0.15	125.02439	289.07193	407.07718	425.08807	161.02455	flavonoids	Crataegus pinnatifida Bge
119	7.352	265.1549072	M+H	$C_{14}H_{20}N_2O_3$	val-phe	264.14763	0.9	72.0808	120.08078	166.08621	265.15485	55.05459	acids/small peptides	
120	7.388	245.1859131	M+H	$C_{12}H_{24}N_2O_3$	Ile-Leu	244.17864	-0.23	86.0964	245.18599	199.18044	132.10191	69.06995	acids/small peptides	
121	7.459	433.1341553	M+H	$C_{18}H_{24}O_{12}$	Licoagroside B	432.12688	0.25	127.03907	85.02847	99.0441	271.06009	103.03902	iridoids	Cornus officinalis Sieb
122	7.49	263.1393127	M+H	$C_{14}H_{18}N_2O_3$	Phe-Pro	262.13204	1.12	120.08077	116.07059	70.06522	263.13895	103.05418	acids/small peptides	
123	7.637	417.1177673	M+H	$C_{21}H_{20}O_9$	Puerarin	416.11057	-0.39	417.11801	297.07574	267.06512	239.07022	399.10742	flavonoids	Pueraria lobata
124	7.694	245.1859131	M+H	$C_{12}H_{24}N_2O_3$	Leu-Ile	244.17864	-0.22	86.09645	245.18616	69.07002	245.10059	132.10194	acids/small peptides	
125	7.706	353.0878296	M-H	$C_{16}H_{18}O_9$	1-Caffeoylquinic acid	354.09511	0.07	191.05623	85.02951	353.08774	93.03454	161.02446	phenylpropanoids	Crataegus pinnatifida Bge
126	7.741	291.0863037	M+H	$C_{15}H_{14}O_6$	Catechin	290.07906	0.09	139.039	123.04406	147.04404	165.05464	291.08691	flavonoids	Astragalus
127	7.746	408.186676	M+NH4	$C_{17}H_{26}O_{10}$	Loganin	390.15278	0.47	179.07034	229.10719	211.09654	109.06491	81.06992	iridoids	[illegible] officinalis [illegible]

No.	RT [min]	Precursor ions	Adduct	Formula	Identification	MW	Diff. ppm	ms/ms fragment ions					Compound Class	Origin plant
128	7.839	172.0980835	M-H	$C_8 H_{15} N O_3$	N-Acetyl-D-alloisoleucine	173.10538	1.1	130.08727	172.09793	93.03456	128.10809	131.09084	acids/small peptides	
129	7.933	337.0930176	M-H	$C_{16} H_{18} O_8$	4-O-p-Coumaroylquinic acid	338.10031	0.42	173.0455	93.03454	163.04004	119.05011	337.09344	phenylpropanoids	pinnatifida
130	7.956	449.1078796	M+H	$C_{21} H_{20} O_{11}$	Orientin	448.10063	0.15	449.1077	299.05484	329.06549	431.09714	283.06006	flavonoids	miltiorrhiza
131	7.965	449.109314	M-H	$C_{21} H_{22} O_{11}$	Hovetrichoside C	450.11669	1.06	449.10889	125.02436	259.06119	269.04562	287.05634	flavonoids	miltiorrhiza
132	7.999	387.201355	M+H	$C_{19} H_{30} O_8$	4S)-4-hydroxy-3,5,5-trimethy -4-[(1E)-3-{[(2R,3R,4S,5S,6R)-3,4,5-trihydroxy-6-(hydroxym ethyl)oxan-2-yl]oxy}but-1-en-1-yl]cyclohex-2-en-1-one	386.19402	-0.12	95.08553	207.13799	67.0544	387.22437	149.09616	flavonoids	Salvia miltiorrhiza Bunge
133	8.001	153.054657	M+H	C8 H8 O3	Vanillin	152.04738	0.2	65.03878	111.04409	125.05975	93.03352	153.05463	flavonoids	miltiorrhiza
134	8.025	377.1452942	M+H	$C_{17} H_{20} N_4 O_6$	Riboflavin (Vitamin B2)	376.13697	-3.5	377.14569	243.08783	172.08693	216.0768	69.03364	/	pinnatifida
135	8.041	359.1334534	M+H	$C_{16} H_{22} O_9$	Sweroside	358.12617	-0.58	197.08084	127.03897	179.07016	111.08046	95.04912	flavonoids	Cornus officinalis Sieb
136	8.075	447.1283875	M+H	$C_{22} H_{22} O_{10}$	3'-Methoxypuerarin	446.12116	-0.3	447.12863	327.0863	297.07581	429.11816	134.03622	flavonoids	Pueraria lobata
137	8.13	549.1598511	M+H	$C_{26} H_{28} O_{13}$	Puerarin apioside	548.15275	-0.44	297.07578	417.11804	267.06516	239.07022	549.16022	flavonoids	Pueraria lobata
138	8.144	342.1698608	M+H	$C_{20} H_{23} N O_4$	(+)-Magnoflorine	341.16258	-0.36	342.17007	58.06546	297.11212	265.08594	282.08862	alkaloids	chinensis
139	8.235	245.1860809	M+H	$C_{12} H_{24} N_2 O_3$	Ile-Ile	244.1788	0.43	86.09643	245.18605	130.04991	84.04446	132.10194	acids/small peptides	
140	8.236	211.1440277	M+H	$C_{11} H_{18} N_2 O_2$	Cyclo(pro-leu)	210.1368	-0.13	211.14412	86.09641	70.06522	136.07574	98.06007	acids/small peptides	
141	8.32	174.1127014	M+H	$C_8 H_{15} N O_3$	N-Acetyl-D-leucine	173.10539	1.15	86.09642	132.10196	128.10703	174.07648	128.07059	acids/small peptides	
142	8.395	163.0401001	M-H	$C_9 H_8 O_3$	2-Hydroxycinnamic acid	164.04743	0.5	119.05016	163.04008	120.05347	93.03461	164.0434	phenylpropanoids	pinnatifida
143	8.434	369.1183167	M+H	$C_{17} H_{20} O_9$	3-O-feruloyl-D-quinic acid	368.11087	0.37	177.05463	145.02843	117.03352	89.03857	149.05978	phenylpropanoids	miltiorrhiza
144	8.501	433.1127625	M+H	$C_{21} H_{20} O_{10}$	Isovitexin	432.10551	-0.31	433.11285	313.07062	283.06012	415.10233	255.06509	flavonoids	Pueraria lobata

No.	RT [min]	Precursor ions	Adduct	Formula	Identification	MW	Diff. ppm	ms/ms fragment ions					Compound class	Origin plant
145	8.561	367.103302	M-H	$C_{17} H_{20} O_9$	4-O-feruloyl-D-quinic acid	368.11058	-0.42	173.0455	93.03452	134.03734	193.05067	137.02432	phenylpropanoids	miltiorrhiza
146	8.707	165.0556641	M-H	$C_9 H_{10} O_3$	3-Phenyllactic acid	166.06295	-0.25	147.04514	165.05563	119.05016	72.99307	121.02946	phenylpropanoids	miltiorrhiza
147	8.745	211.1440277	M+H	$C_{11} H_{18} N_2 O_2$	Cyclo(leucylprolyl)	210.13677	-0.29	211.1441	70.06524	86.09643	98.06008	114.09142	acids/small peptides	
148	8.767	417.1177673	M+H	$C_{21} H_{20} O_9$	Daidzin	416.11059	-0.33	255.06526	199.07542	137.02344	181.06476	91.05426	flavonoids	Pueraria lobata
149	8.79	785.2515259	M-H	$C_{35} H_{46} O_{20}$	Purpureaside C	786.25868	0.56	161.02444	785.25171	133.0295	135.04529	59.01382	flavonoids	Cornus officinalis Sieb
150	8.893	755.2050171	M-H	$C_{33} H_{40} O_{20}$	3-O-rutinoside-(1->2)-O-rha	756.2121	1.06	300.02771	271.02493	755.20502	255.03001	243.02992	flavonoids	Cornus officinalis Sieb
151	8.894	279.1706543	M+H	$C_{15} H_{22} N_2 O_3$	L-Isoleucyl-D-phenylalanine	278.16337	1.17	86.09645	69.07002	279.17047	120.08089	166.08636	acids/small peptides	
152	8.9	337.0931396	M-H	$C_{16} H_{18} O_8$	5-O-p-Coumaroylquinic acid	338.10043	0.76	191.05621	111.00875	85.0295	337.09357	173.00916	phenylpropanoids	miltiorrhiza
153	8.954	449.1077881	M+H	$C_{21} H_{20} O_{11}$	Cynaroside	448.10066	0.22	287.05499	153.0182	241.04942	269.0444	213.0545	flavonoids	Cornus officinalis Sieb
154	8.967	403.1245422	M-H	$C_{17} H_{24} O_{11}$	Secoxyloganin	404.1319	0.1	121.02949	59.01383	71.01381	89.02443	371.09818	iridoids	Cornus officinalis Sieb
155	8.973	325.0928955	M-H	$C_{15} H_{18} O_8$	2Z)-2-(beta-D-Glucopyranosy loxy)-3-phenylacrylic acid	326.10013	-0.12	119.0502	325.09341	163.03976	101.0397	222.05348	phenylpropanoids	miltiorrhiza
156	9.002	275.0913086	M+H	$C_{15} H_{14} O_5$	Phloretin	274.08403	-0.35	107.04918	137.02338	229.08601	119.0492	257.08096	flavonoids	Cornus officinalis Sieb
157	9.067	539.2137451	M-H	$C_{26} H_{36} O_{12}$	Sonchifolignan A	540.22104	0.67	491.19269	539.21429	150.03215	165.05568	149.02435	phenylpropanoids	miltiorrhiza
158	9.186	173.081955	M-H	$C_8 H_{14} O_4$	Suberic acid	174.08924	0.17	111.08149	173.08186	83.05025	93.03454	129.09206	organic acids	miltiorrhiza
159	9.192	328.15448	M+H	$C_{19} H_{21} N O_4$	Boldine	327.1472	0.44	297.11227	265.08606	328.15399	205.06487	233.05983	alkaloids	chinensis
160	9.262	447.1283875	M+H	$C_{22} H_{22} O_{10}$	Glycitin	446.12121	-0.19	285.07578	270.05237	253.04944	213.05446	137.02332	flavonoids	Cornus officinalis Sieb
161	9.275	431.0983582	M-H	$C_{21} H_{20} O_{10}$	Vitexin	432.10559	-0.13	311.0564	431.09839	283.06122	133.02931	135.04515	flavonoids	pinnatifida
162	9.294	208.0969086	M+H	$C_{11} H_{13} N O_3$	N-Acetyl-L-phenylalanine	207.08964	0.47	120.08078	166.0862	162.09125	208.09569	103.05431	acids/small peptides	
163	9.315	539.213501	M-H	$C_{26} H_{36} O_{12}$	oxy-3-methoxyphenyl)propar 2-yl)oxy)-3-methoxyphenyl)p	540.22088	0.38	165.05563	491.19269	150.03217	195.06627	149.02432	flavonoids	pinnatifida
164	9.359	318.1812744	M+H	$C_{17} H_{23} N_3 O_3$	Ile-Trp	317.174	0.18	86.0964	205.09721	318.18152	188.07059	318.22186	acids/small peptides	
165	9.399	302.2073975	M+H	$C_{14} H_{27} N_3 O_4$	ile-gly-ile	301.20013	-0.08	86.09636	143.11783	171.11263	75.05528	132.10184	acids/small peptides	
166	9.427	609.1468506	M-H	$C_{27} H_{30} O_{16}$	Rutin	610.15382	0.71	300.02777	609.14691	271.02502	255.03055	565.13483	flavonoids	pinnatifida
167	9.442	579.1723633	M-H	$C_{27} H_{32} O_{14}$	Narirutin	580.17932	0.19	271.0614	579.17242	151.00365	119.05022	107.01388	flavonoids	pinnatifida
168	9.507	413.1454773	M-H	$C_{19} H_{26} O_{10}$	-O-(4-carboxy-3-hydroxy-3-methylbutanoyl)hexopyranosic	414.15276	0.39	99.04514	57.03454	59.01384	125.02438	101.02437	flavonoids	pinnatifida

No.	RT [min]	Precursor ions	Adduct	Formula	Identification	MW	Diff. ppm	ms/ms fragment ions					Compound Class	Origin plant
169	9.566	300.9989319	M-H	$C_{14}H_{6}O_{8}$	Ellagic acid	302.00622	-0.16	300.99905	283.99643	229.01419	299.99142	185.02452	phenols	miltiorrhiza
170	9.587	417.1177673	M+H	$C_{21}H_{20}O_{9}$	daidzein-6-C-glucoside	416.11061	-0.3	417.1178	297.07562	267.06506	399.10727	239.07013	flavonoids	Pueraria lobata
171	9.591	279.1706543	M+H	$C_{15}H_{22}N_{2}O_{3}$	Phe-leu	278.16336	1.15	86.09639	120.08079	279.17038	166.08623	149.02335	acids/small peptides	
172	9.664	302.2073975	M+H	$C_{14}H_{27}N_{3}O_{4}$	leu-gly-leu	301.20013	-0.07	86.09647	171.11287	189.12346	132.10196	302.20737	acids/small peptides	
173	9.729	417.1177673	M+H	$C_{21}H_{20}O_{9}$	Daidzein 4'-O-glucoside	416.11055	-0.43	255.06503	417.11786	199.07524	85.02837	137.02332	flavonoids	Pueraria lobata
174	9.795	417.1193542	M-H	$C_{21}H_{22}O_{9}$	Liquiritin	418.12667	0.69	255.06648	119.05019	135.0088	153.01935	91.01894	flavonoids	Pueraria lobata
175	9.851	245.0931396	M-H	$C_{13}H_{14}N_{2}O_{3}$	N-Acetyl-DL-tryptophan	246.10042	-0.11	245.09317	74.02474	203.08276	116.05046	98.02473	acids/small peptides	
176	9.864	447.1283875	M+H	$C_{22}H_{22}O_{10}$	Calycosin-7-O-β-D-glucoside	446.12111	-0.42	285.07578	270.05219	137.02328	213.05438	225.05461	flavonoids	Pueraria lobata
177	9.972	465.1028442	M+H	$C_{21}H_{20}O_{12}$	Quercetin-3β-D-glucoside	464.09564	0.35	303.05002	61.02871	85.02844	153.0182	229.04964	flavonoids	taegus pinnatifida
178	10.052	417.1177673	M+H	$C_{21}H_{20}O_{9}$	daidzein-5-C-glucoside	416.1106	-0.31	417.11823	297.07587	267.06522	239.07028	399.10757	flavonoids	Pueraria lobata
179	10.064	257.08078	M+H	$C_{15}H_{12}O_{4}$	Liquiritigenin	256.07354	-0.08	137.02333	257.08078	147.04399	81.03351	119.04916	flavonoids	Pueraria lobata
180	10.094	197.1173553	M+H	$C_{11}H_{16}O_{3}$	5-(4-Hydroxypentyl)-1,3-benzenediol	196.11008	0.7	197.11717	179.10663	133.10121	107.08553	135.11682	phenols	miltiorrhiza
181	10.12	477.0674744	M-H	$C_{21}H_{18}O_{13}$	Quercetin 3-O-β -D-Glucuronide	478.07485	0.23	301.03558	151.00365	477.06769	178.99858	121.02943	flavonoids	pinnatifida
182	10.151	465.1028442	M+H	$C_{21}H_{20}O_{12}$	Hyperoside	464.09563	0.33	303.04993	85.02837	153.01797	465.13202	69.03356	flavonoids	pinnatifida Bge/Cornus
183	10.191	565.1552124	M+H	$C_{26}H_{28}O_{14}$	Apiin	564.14794	0.05	271.06009	433.11304	153.01825	91.05421	215.06987	flavonoids	Cornus officinalis Sieb
184	10.232	313.1549072	M+H	$C_{18}H_{20}N_{2}O_{3}$	Di-L-phenylalanine	312.14766	0.85	120.08085	103.05427	313.15283	166.08636	91.05429	acids/small peptides	Astragalus
185	10.37	433.1127625	M+H	$C_{21}H_{20}O_{10}$	Genistin	432.1056	-0.1	271.06003	153.01817	91.05421	215.07022	433.22925	flavonoids	Pueraria lobata
186	10.397	322.1072388	M+H	$C_{19}H_{15}NO_{4}$	Berberrubine	321.09996	-0.45	322.10739	307.08392	250.08626	279.08908	278.08127	alkaloids	chinensis
187	10.46	503.1181641	M+H	$C_{24}H_{22}O_{12}$	malonyldaidzin	502.11089	-0.47	255.06523	199.07536	137.02339	181.06471	91.05425	flavonoids	Pueraria lobata
188	10.48	284.1394653	M+H	$C_{16}H_{17}N_{3}O_{2}$	cyclo(tryptophyl-prolyl)	283.13219	0.4	130.0652	284.1394	170.05997	70.06525	132.08078	acids/small peptides	

No.	RT [min]	Precursor ions	Adduct	Formula	Identification	MW	Diff. ppm	ms/ms fragment ions					Compound Class	Origin plant
189	10.572	479.1551208	M+H	$C_{23}H_{26}O_{11}$	Calceolarioside B	478.14779	0.58	163.03897	135.04405	89.03857	117.0335	325.09192	phenylpropanods	miltiorrhiza
190	10.861	356.1859131	M+H	$C_{21}H_{25}NO_4$	Tetrahydropalmatine	355.17864	0.78	192.10187	356.1861	177.07846	356.14795	149.08362	alkaloids	chinensis
191	10.882	463.1236267	M+H	$C_{22}H_{22}O_{11}$	Tectoridin	462.11635	0.3	301.07062	286.04712	269.0444	229.04955	153.01816	flavonoids	pinnatifida
192	11.056	519.1873779	M-H	$C_{26}H_{32}O_{11}$	Pinoresinol 4-O-glucoside	520.19466	0.39	151.04002	357.13455	136.01653	342.11108	121.02928	flavonoids	pinnatifida
193	11.108	479.1549988	M+H	$C_{23}H_{26}O_{11}$	Calceolarioside A	478.14765	0.3	163.03897	135.04404	89.03857	117.03355	145.0284	phenylpropanods	miltiorrhiza
194	11.112	623.1984863	M-H	$C_{29}H_{36}O_{15}$	Isoacteoside	624.20574	0.5	161.02449	623.19922	133.02951	135.04515	113.02451	saponins	Bunge
195	11.175	283.0612183	M-H	$C_{16}H_{12}O_5$	Biochanin A	284.06846	-0.04	283.06134	268.03787	211.04012	239.03543	240.04276	flavonoids	pinnatifida
196	11.25	521.2029419	M-H	$C_{26}H_{34}O_{11}$	yl)-3-(hydroxymethyl)-7-methoxy -2,3-dihydro-1-benzofuran-5-yl	522.21029	0.34	491.19284	503.19272	59.01384	71.01384	329.1394	flavonoids	Crataegus pinnatifida
197	11.307	579.2088013	M-H	$C_{28}H_{36}O_{13}$	Acanthoside B	580.21614	0.95	417.15573	181.05069	166.02708	387.10849	151.00357	phenylpropanods	miltiorrhiza
198	11.429	336.1227722	M+H	$C_{20}H_{17}NO_4$	Epiberberine	335.1155	-0.78	336.12283	320.09164	292.09665	262.08618	290.08102	alkaloids	chinensis
199	11.443	435.1287537	M+H	$C_{21}H_{22}O_{10}$	Prunin	434.1214	0.24	273.07571	153.0182	147.04404	119.04919	91.05422	flavonoids	miltiorrhiza
200	11.493	463.1230774	M+H	$C_{22}H_{22}O_{11}$	Diosmetin-7-O-β -D-glucopyranoside	462.1158	-0.89	301.07071	286.0473	229.04959	153.0182	241.04955	flavonoids	miltiorrhiza
201	11.516	187.0976105	M-H	$C_9H_{16}O_4$	Azelaic acid	188.10488	0.11	125.09715	187.09766	123.08163	126.10057	57.03451	organic acids	miltiorrhiza
202	11.558	338.1385193	M+H	$C_{20}H_{19}NO_4$	Dihydroberberine	337.13124	-0.49	338.13855	322.1073	279.08902	294.11218	265.07349	alkaloids	chinensis
203	11.624	195.0651703	M+H	$C_{10}H_{10}O_4$	Ferulic acid	194.05794	0.15	177.05463	145.02843	117.03349	149.05975	89.03856	phenylpropanods	chinensis
204	11.661	541.15625	M-H	$C_{24}H_{30}O_{14}$	Cornuside	542.16346	-0.18	169.01427	125.02437	541.1568	81.03455	379.10434	iridoids	Cornus officinalis Sieb
205	11.688	287.0912476	M+H	$C_{16}H_{14}O_5$	5-O-Methylnaringenin	286.08395	-0.62	167.03387	287.09134	91.05422	153.05461	147.04396	flavonoids	miltiorrhiza
206	11.803	338.1385193	M+H	$C_{20}H_{19}NO_4$	Jatrorrhizine	337.13124	-0.49	338.13855	322.10736	279.08896	294.11224	265.07336	alkaloids	chinensis
207	11.835	459.1283264	M+H	$C_{23}H_{22}O_{10}$	6-O-Acetyldaidzin	458.12105	-0.54	255.06532	199.07539	137.02344	91.0542	181.0648	flavonoids	Pueraria lobata
208	11.862	195.0651703	M+H	$C_{10}H_{10}O_4$	Isoferulic acid	194.05802	0.55	177.0547	89.03862	117.03356	149.05984	163.03905	phenylpropanods	miltiorrhiza
209	11.865	181.0497131	M+H	$C_9H_8O_4$	Caffeic acid	180.04225	-0.04	163.03893	135.04404	145.02844	117.03349	89.03855	phenylpropanods	Bunge/Astragalus/Crataegu

No.	RT [min]	Precursor ions	Adduct	Formula	Identification	MW	Diff. ppm	ms/ms fragment ions					Compound Class	Origin plant
210	11.866	359.0773926	M-H	$C_{18}H_{16}O_8$	Rosmarinic acid	360.08462	0.28	161.02443	197.04559	135.04517	72.9931	133.02951	phenylpropanoids	miltiorrhiza
211	12.16	537.1039429	M-H	$C_{27}H_{22}O_{12}$	Lithospermic acid	538.11127	0.26	295.06131	109.0294	185.02446	135.0451	159.0452	phenylpropanoids	miltiorrhiza
212	12.232	489.1393127	M+H	$C_{24}H_{24}O_{11}$	6''-O-Acetylglycitin	488.13204	0.36	285.07584	270.05237	253.04948	213.05458	137.02335	flavonoids	Pueraria lobata
213	12.432	475.1595459	M+H	$C_{24}H_{26}O_{10}$	2-[2-(4-Hydroxybenzyl)-5-oxo-2 5-dihydro-3-furanyl]-5-methoxy phenyl beta-D-glucopyranoside	474.15248	-0.25	107.04915	313.10706	295.0965	267.10168	85.0284	flavonoids	Salvia miltiorrhiza Bunge
214	12.529	336.1227722	M+H	$C_{20}H_{17}NO_4$	Berberine	335.1155	-0.78	336.12311	320.09192	292.09692	278.08133	318.07626	alkaloids	chinensis
215	12.546	284.1279297	M+H	$C_{17}H_{17}NO_3$	N-p-Coumaroyltyramine	283.12067	-0.6	147.04407	121.06484	284.12814	91.05426	119.04922	phenylpropanoids	Ophiopogon japonicus
216	12.709	717.1464844	M-H	$C_{36}H_{30}O_{16}$	Salvianolic acid B	718.15362	0.33	321.04053	519.09344	109.02946	339.05112	295.06131	phenylpropanoids	miltiorrhiza
217	12.88	255.0652161	M+H	$C_{15}H_{10}O_4$	Daidzein	254.05797	0.23	255.06509	199.0753	137.02332	91.0542	181.06461	flavonoids	Pueraria lobata
218	12.981	159.1026764	M-H	$C_8H_{16}O_3$	3-Hydroxyoctanoic acid	160.10996	0.11	59.01382	159.10268	159.0298	73.02939	129.01939	organic acids	Cornus officinalis Sieb
219	12.996	314.1389771	M+H	$C_{18}H_{19}NO_4$	Moupinamide	313.13169	0.89	177.05461	121.06481	314.13885	145.02841	117.03349	alkaloids	chinensis
220	13.005	285.0406189	M-H	$C_{15}H_{10}O_6$	Luteolin	286.04776	0.09	285.04053	217.05058	175.04002	133.02946	105.01924	flavonoids	Astragalus
221	13.037	419.1338806	M+H	$C_{21}H_{22}O_9$	Dihydrodaidzin	418.12659	0.49	257.08093	137.02341	147.04408	119.04925	81.03356	flavonoids	Pueraria lobata
222	13.083	257.0809021	M+H	$C_{15}H_{12}O_4$	Isoliquiritigenin	256.07362	0.23	257.08099	137.02338	147.04405	81.03354	91.05424	flavonoids	Pueraria lobata
223	13.339	285.0759583	M+H	$C_{16}H_{12}O_5$	Glycitein	284.06864	0.57	285.07565	270.05225	137.0233	213.05452	253.04939	flavonoids	Pueraria lobata
224	13.342	407.1347961	M-H	$C_{20}H_{24}O_9$	Tinnevellin glucoside	408.14199	-0.1	245.08186	230.05843	215.03508	407.13251	187.03995	phenols	miltiorrhiza
225	13.394	717.1464844	M-H	$C_{36}H_{30}O_{16}$	Salvianolic acid E	718.15374	0.49	321.04053	519.09357	109.02943	185.02443	339.05109	phenylpropanoids	miltiorrhiza
226	13.482	197.0455017	M-H	$C_9H_{10}O_5$	Syringic acid	198.05298	0.77	182.02217	197.04558	123.00878	166.99854	72.99311	phenols	miltiorrhiza
227	13.524	475.1238708	M+H	$C_{23}H_{22}O_{11}$	6-O-Acetylgenistin	474.11649	0.59	271.06006	153.01817	91.05421	215.07025	149.0233	flavonoids	Pueraria lobata
228	13.58	493.1141663	M-H	$C_{26}H_{22}O_{10}$	Salvianolic acid A	494.12152	0.45	295.0611	109.0294	185.02438	135.0451	159.04507	phenylpropanoids	miltiorrhiza
229	13.678	463.1603394	M+H	$C_{23}H_{26}O_{10}$	Methylnissolin-3-O-glucoside	462.1531	1.1	167.07025	301.10709	134.03622	152.0468	106.04133	phenols	Astragalus

No.	RT [min]	Precursor ions	Adduct	Formula	Identification	MW	Diff. ppm	ms/ms fragment ions					Compound Class	Origin plant
230	13.688	201.1134033	M-H	$C_{10}H_{18}O_4$	Sebacic acid	202.12068	0.87	201.11331	139.1129	183.10269	111.02	202.11624	organic acids	Cornus officinalis Sieb
231	13.741	461.1443787	M+H	$C_{23}H_{24}O_{10}$	',6-Dimethoxyisoflavone-7-O-β-D-glucopyranoside	460.1371	0.34	299.09143	284.0679	256.07278	241.04924	161.05957	flavonoids	pinnatifida
232	13.749	301.0354309	M-H	$C_{15}H_{10}O_7$	Quercetin	302.04275	0.33	301.03549	151.00372	301.18134	178.99867	121.02949	flavonoids	pinnatifida Bge/Cornus
233	13.759	285.0759583	M+H	$C_{16}H_{12}O_5$	Calycosin	284.06864	0.58	285.07568	270.05222	213.05452	137.0233	253.04942	flavonoids	Astragalus
234	13.799	334.1077271	M+H	$C_{20}H_{15}NO_4$	Dihydrosanguinarine	333.10045	1.03	334.10736	276.06561	318.0762	290.08124	304.06049	alkaloids	chinensis
235	13.967	651.2298584	M-H	$C_{31}H_{40}O_{15}$	Martynoside	652.23718	0.7	651.22998	175.04007	160.0166	134.03737	193.0507	ohenylpropar oids	miltiorrhiza
236	13.987	593.1866455	M+H	$C_{28}H_{32}O_{14}$	Fortunellin	592.17937	0.28	285.07578	242.0573	447.12878	270.05222	153.01814	flavonoids	Cornus officinalis Sieb
237	14.18	463.1611633	M-H	$C_{23}H_{28}O_{10}$	somucronulatol 7-O-glucoside	464.16844	0.41	301.10822	121.02946	135.04517	271.0614	286.08484	flavonoids	Astragalus
238	14.192	315.0863953	M+H	$C_{17}H_{14}O_6$	',7-dihydroxy-4',6-dimethoxyi oflavone	314.07912	0.26	315.08633	300.06281	243.06519	167.03413	184.03566	flavonoids	miltiorrhiza
239	14.283	463.1236267	M+H	$C_{22}H_{22}O_{11}$	Kaempferide 3-O-beta-D-glucopyranoside	462.11644	0.49	301.07074	231.06529	167.03392	85.0284	203.07033	flavonoids	Astragalus
240	14.43	517.1340942	M+H	$C_{25}H_{24}O_{12}$	formononetin 7-O-glucoside-6''-O-malonate	516.12685	0.15	269.08102	197.05983	253.04968	213.09113	254.05737	flavonoids	miltiorrhiza
241	15.102	269.0455933	M-H	$C_{15}H_{10}O_5$	Genistein	270.05286	0.14	269.04559	133.02943	63.02398	224.04774	181.06627	flavonoids	Pueraria lobata
242	15.197	447.1288147	M+H	$C_{22}H_{22}O_{10}$	Isoprunetin 7-O-glucoside	446.12151	0.49	285.07578	213.05455	270.05228	242.05724	170.02098	flavonoids	Pueraria lobata
243	16.089	327.2177124	M-H	$C_{18}H_{32}O_5$	(11E,15Z)-9,10,13-Trihydroxy-1 1,15-octadecadienoic acid	328.22502	0.13	327.21793	171.10269	85.0295	211.13425	137.09731	organic acids	pinnatifida
244	16.214	327.2177124	M-H	$C_{18}H_{32}O_5$	(10E,15Z)-9,12,13-Trihydroxy-1 0,15-octadecadienoic acid	328.22501	0.12	327.2179	171.10269	211.13405	229.14455	85.02949	organic acids	pinnatifida
245	16.438	799.4804688	M-H	$C_{42}H_{72}O_{14}$	Ginsenoside Rg1	800.4882	-3.01	799.48627	71.01387	59.01385	101.0244	85.02954	saponins	Panax ginseng
246	16.491	499.2072754	M+H	$C_{26}H_{30}N_2O_8$	Vincosamide	498.2	-0.44	171.09174	337.15479	144.08087	267.11304	154.06519	alkaloids	chinensis
247	16.88	769.4695435	M-H	$C_{41}H_{70}O_{13}$	20(R)-Notoginsenoside R2	770.47682	-3.26	769.47534	71.01384	637.43256	59.01383	475.37946	saponins	Panax ginseng
248	16.935	1211.641968	M+H	$C_{58}H_{98}O_{26}$	Ginsenoside Ra1	1210.63426	-0.3	85.02841	295.10251	325.11313	69.03362	145.04959	saponins	Panax ginseng
249	17.109	947.5558472	M+H	$C_{48}H_{82}O_{18}$	Ginsenoside Re	946.54875	-1.45	85.02842	325.11301	145.04956	127.03905	163.06007	saponins	Panax ginseng

No.	RT [min]	Precursor ions	Adduct	Formula	Identification	MW	Diff. ppm	ms/ms fragment ions					Compound Class	Origin plant
250	17.11	443.38797	M+H	$C_{30}H_{50}O_2$	Erythrodiol	442.38069	-0.88	443.38858	81.06992	95.08556	107.08553	119.08562	/	Bunge
251	17.185	293.210968	M+H	$C_{18}H_{28}O_3$	12-oxo Phytodienoic Acid	292.20369	-0.52	275.20065	293.21091	81.06988	79.05425	67.05438	organic acids	Cornus officinalis Sieb
252	17.224	329.2334595	M-H	$C_{18}H_{34}O_5$	(9Z)-5,8,11-Trihydroxy-9-oct adecenoic acid	330.24074	0.34	329.23355	171.10266	211.13406	139.11281	229.14427	organic acids	Crataegus pinnatifida Bge
253	17.279	269.080719	M+H	$C_{16}H_{12}O_4$	Formononetin	268.07348	-0.3	269.08075	197.05965	253.04947	237.05455	254.05742	flavonoids	Pueraria lobata
254	17.424	683.4378052	M+FA-H	$C_{36}H_{62}O_9$	Ginsenoside F1	684.44508	0.3	683.4386	637.43164	97.05043	97.05717	475.37567	saponins	Panax ginseng
255	17.734	457.3677063	M+H	$C_{30}H_{48}O_3$	Ursolic acid	456.36044	0.2	411.36237	95.08562	107.08551	93.0699	81.06995	/	miltiorrhiza
256	17.882	264.1593933	M+H	$C_{15}H_{21}NO_3$	N-(4-Methylpentanoyl)pheny alanine	263.15213	-0.05	120.0808	166.08626	103.05423	71.08561	99.08044	acids/small peptides	
257	18.029	352.1178589	M+H	$C_{20}H_{17}NO_5$	Oxyberberine	351.11058	-0.26	352.11765	308.09167	280.09665	324.12274	336.08633	alkaloids	Coptis chinensi Franch
258	18.189	303.1227417	M+H	$C_{17}H_{18}O_5$	Isomucronulatol	302.11547	0.14	123.04405	167.0703	133.06483	118.04131	95.04913	flavonoids	Astragalus
259	18.208	785.4678955	M+H	$C_{41}H_{68}O_{14}$	Astragaloside IV	784.46055	-0.45	143.10674	125.09629	107.08559	437.3421	71.04926	saponins	Astragalus
260	18.412	785.4678955	M+H	$C_{41}H_{68}O_{14}$	Astragaloside III	784.46055	-1.2	143.10677	125.09618	107.08558	437.3418	71.04926	saponins	Astragalus
261	18.801	443.3882751	M+H	$C_{30}H_{50}O_2$	Betulin	442.381	-0.19	443.38843	81.06992	95.08556	107.08552	119.08564	/	Bunge
262	18.803	947.5563354	M+H	$C_{48}H_{82}O_{18}$	Ginsenoside Rd	946.54965	-0.49	85.02847	145.04962	443.38828	425.3783	407.36758	saponins	Panax ginseng
263	18.821	295.2265625	M+H	$C_{18}H_{30}O_3$	13(S)-HOTrE	294.21929	-0.71	295.22653	277.21631	67.05444	251.10683	81.06995	organic acids	
264	19.284	403.1387024	M+H	$C_{21}H_{22}O_8$	Nobiletin	402.13143	-0.1	403.13882	373.09174	105.03354	183.0289	127.03897	flavonoids	Cornus officinalis Sieb
265	19.447	827.4780273	M+H	$C_{43}H_{70}O_{15}$	Astragaloside II	826.4715	-1.3	325.11337	163.06021	85.0284	157.04953	115.03897	saponins	Astragalus
266	19.494	947.5570679	M+H	$C_{48}H_{82}O_{18}$	Gypenoside XVII	946.54999	-0.13	85.02841	145.04958	163.06003	325.11307	407.36691	saponins	Ophiopogon japonicus
267	20.023	961.5386353	M-H	$C_{48}H_{82}O_{19}$	Gypenoside XLVI	962.54591	0.92	59.01382	71.01383	89.02441	149.04543	131.03493	saponins	Ophiopogon japonicus
268	20.034	955.4911499	M-H	$C_{48}H_{76}O_{19}$	Ginsenoside Ro	956.4983	0.23	72.99306	71.01384	955.4931	113.02436	85.02947	saponins	Panax ginseng
269	20.184	943.5254517	M+H	$C_{48}H_{78}O_{18}$	Soyasaponin I	942.51891	0.1	441.37305	423.36221	85.02843	95.08556	141.01817	saponins	Pueraria lobata
270	20.255	283.0612183	M-H	$C_{16}H_{12}O_5$	Texasin	284.06858	0.38	283.06128	268.03787	267.03006	239.03503	211.04021	flavonoids	Cornus officinalis Sieb

No.	RT [min]	Precursor ions	Adduct	Formula	Identification	MW	Diff. ppm	ms/ms fragment ions					Compound class	Origin plant
271	20.416	961.5388184	M-H	$C_{48}H_{82}O_{19}$	20-Glucoginsenoside Rf	962.54609	1.11	59.01383	71.01385	89.02444	149.04541	915.53296	saponins	Panax ginseng
272	20.559	809.4334717	M-H	$C_{42}H_{66}O_{15}$	Rivaloside B	810.44023	0.08	809.4342	113.02441	85.02951	71.01386	351.05731	saponins	Ophiopogon japonicus
273	20.84	373.1279602	M+H	$C_{20}H_{20}O_{7}$	Tangeritin	372.12068	-0.59	373.12827	343.08112	183.02892	297.07559	135.04407	flavonoids	Cornus officinalis Sieb
274	21.376	311.2227478	M-H	$C_{18}H_{32}O_{4}$	(+/-)9-HpODE	312.22998	-0.27	311.22302	223.17036	87.04512	293.21268	57.03451	organic acids	
275	21.692	941.5098877	M+H	$C_{48}H_{76}O_{18}$	Dehydrosoyasaponin I	940.50333	0.17	439.35718	85.02839	421.34644	95.08551	119.0856	saponins	Panax ginseng
276	22.426	869.4885254	M+H	$C_{45}H_{72}O_{16}$	Isoastragaloside I	868.482	-1.6	97.02842	143.10664	139.03899	69.03359	217.07066	saponins	Astragalus
277	22.545	279.1016235	M+H	$C_{18}H_{14}O_{3}$	Dihydroisotanshinone I	278.09434	0.15	279.10175	233.09631	205.10136	190.07774	261.09125	phenols	miltiorrhiza
278	22.728	819.4675903	M+Cl	$C_{42}H_{72}O_{13}$	Rg3	820.47487	-2.5	819.4682	783.49139	71.01385	101.02438	85.02952	saponins	Panax ginseng
279	23.053	869.4889526	M+H	$C_{45}H_{72}O_{16}$	Isoastragaloside isomer	868.48169	-0.4	143.10667	69.03361	125.09612	157.04959	71.0492	saponins	Astragalus
280	23.422	631.3853149	M-H	$C_{36}H_{56}O_{9}$	Calenduloside E	632.39259	0.25	631.38562	75.00873	85.02948	113.02441	455.35336	saponins	Ophiopogon japonicus
281	23.527	295.1329956	M+H	$C_{19}H_{18}O_{3}$	Tanshinone Iia	294.12572	0.42	295.13306	280.10947	249.12761	191.08562	225.12729	/	miltiorrhiza
282	23.659	855.4733887	M+H	$C_{44}H_{70}O_{16}$	Ophiopogonin D	854.46613	-0.3	85.02845	413.30569	395.29486	71.04926	269.19012	saponins	Ophiopogon japonicus
283	23.714	485.327179	M-H	$C_{30}H_{46}O_{5}$	2,19-Dihydroxy-3-oxo-12-urs en-28-oic acid	486.33455	0.05	485.32773	467.31708	425.30621	423.32864	71.05025	/	Bunge
284	24.102	371.1124573	M+H	$C_{20}H_{18}O_{7}$	Meridinol	370.10518	-0.19	135.04405	371.11261	79.05425	95.04916	105.03356	phenylpropanoids	miltiorrhiza
285	24.25	297.1485291	M+H	$C_{19}H_{20}O_{3}$	Cryptotanshinone	296.14125	0.01	297.14862	251.14304	254.09386	282.12518	279.13794	/	miltiorrhiza
286	24.3	277.0860596	M+H	$C_{18}H_{12}O_{3}$	Tanshinon I	276.07874	0.34	277.08603	178.0777	249.091	193.1012	202.07774	/	miltiorrhiza
287	24.874	279.1017761	M+H	$C_{18}H_{14}O_{3}$	Dihydroisotanshinone II	278.09448	0.68	261.09116	190.07771	233.09622	205.10127	189.06987	/	miltiorrhiza
288	25.112	295.2268982	M+H	$C_{18}H_{30}O_{3}$	9-Oxo-10(E),12(E)-octadecadenoic acid	294.21962	0.43	277.21631	81.03353	67.0544	81.06989	79.05424	organic acids	pinnatifida
289	25.648	295.1329956	M+H	$C_{19}H_{18}O_{3}$	Tanshinone IIA	294.1257	0.36	295.13315	249.1275	277.12247	191.08549	206.10921	/	miltiorrhiza
290	26.194	469.3318787	M-H	$C_{30}H_{46}O_{4}$	18-β-Glycyrrhetinic acid	470.33933	-0.6	469.33243	68.81814	299.99152	423.32745	116.92844	/	Panax ginseng
